# The Development and Optimization of Extrusion-Based 3D Food Printing Inks Using Composite Starch Gels Enriched with Various Proteins and Hydrocolloids

**DOI:** 10.3390/gels11080574

**Published:** 2025-07-23

**Authors:** Evgenia N. Nikolaou, Eftychios Apostolidis, Eirini K. Nikolidaki, Evangelia D. Karvela, Athena Stergiou, Thomas Kourtis, Vaios T. Karathanos

**Affiliations:** Department of Science of Dietetics-Nutrition, Harokopion University, 70, El. Venizelou, Kallithea, 17671 Athens, Greece; apostolidis.eft@gmail.com (E.A.); irenenikolidaki@hotmail.com (E.K.N.); ekarvela@hua.gr (E.D.K.); athster@hua.gr (A.S.); kourtistommy@gmail.com (T.K.); vkarath@hua.gr (V.T.K.)

**Keywords:** 3D food printing, composite starch gels, printability, computational fluid dynamics

## Abstract

This study presents a comprehensive evaluation of starch-based gel formulations enriched with proteins and hydrocolloids for extrusion-based 3D food printing (3DFP). Food inks were prepared using corn or potato starch, protein concentrates (fava, whey, rice, pea and soya), and hydrocolloids (κ-carrageenan, arabic gum, xanthan gum, and carboxy methylcellulose). Their rheological, mechanical, and textural properties were systematically analyzed to assess printability. Among all formulations, those containing κ-carrageenan consistently demonstrated superior viscoelastic behavior (G′ > 4000 Pa), optimal tan δ values (0.096–0.169), and yield stress conducive to stable extrusion. These inks also achieved high structural fidelity (93–96% accuracy) and favourable textural attributes such as increased hardness and chewiness. Computational Fluid Dynamics (CFD) simulations further validated the inks’ performances by linking pressure and velocity profiles with rheological parameters. FTIR analysis revealed that gel strengthening was primarily driven by non-covalent interactions, such as hydrogen bonding and electrostatic effects. The integration of empirical measurements and simulation provided a robust framework for evaluating and optimizing printable food gels. These findings contribute to the advancement of personalized and functional 3D-printed foods through data-driven formulation design.

## 1. Introduction

Three-dimensional (3D) printing is an emerging technology gaining increasing attention across diverse fields, such as food production, owing to its versatility, cost-efficiency, and capacity for design customization [1]. In the context of food applications, 3D food printing (3DFP) offers significant advantages over traditional food processing methods, enabling the rapid fabrication of complex, customizable structures tailored to specific nutritional and sensory needs [2]. For instance, plant-based proteins such as soy or pea protein isolates can be precisely structured through 3DFP to replicate the fibrous texture of animal meat, while allowing for the incorporation of specific micronutrients or functional ingredients to enhance the nutritional profile of the final product [3].

Among the various approaches to 3DFP, extrusion-based printing is the most commonly employed due to its simplicity, affordability, and suitability for soft food materials. This process typically involves four key steps: (i) food ink preparation, (ii) material extrusion, (iii) deposition onto the printing platform, and (iv) 3D structure formation [4,5]. However, a major challenge in this method lies in developing food inks with optimal formulation and rheological properties to ensure both the printability and structural integrity of the final construct.

Starch has been widely explored as a base material for 3D food printing (3DFP) due to its natural abundance, its ability to develop a gel-like structure upon heating, and its favorable shear-thinning and viscoelastic properties in the gelatinized state. [6]. It is primarily composed of two polysaccharides: amylose and amylopectin [7]. Amylose is a primarily linear polymer composed of glucose units linked by α-1,4-glycosidic bonds, whereas amylopectin is a much larger, highly branched molecule due to the presence of both α-1,4- and α-1,6-glycosidic linkages [7]. Within starch granules, these two components organize into alternating crystalline and amorphous layers arranged in a radial pattern. The relative proportions and structural characteristics of amylose and amylopectin vary depending on the botanical source, leading to differences in granule shape, size, and internal architecture [8]. These botanical variations are particularly important when evaluating starches for 3D food printing applications. Different starch sources can significantly affect key functional properties such as gelatinization, viscosity, structural integrity (crystallinity), and printability. Understanding how starch composition influences these behaviors is essential for optimizing formulations and achieving consistent, high-quality printed food products.

As a printed material, starch alone does not constitute a suitable food ink, often necessitating the incorporation of additional components—such as proteins and hydrocolloids—to enhance its mechanical properties, minimize syneresis, and improve printability [9,10]. Protein concentrates from alternative sources—such as legumes (soy, pea, fava bean), cereals (rice, corn), and microbial or plant leaves—are being increasingly investigated for their nutritional and functional roles in 3DFP formulations [11,12]. Likewise, hydrocolloids, including xanthan gum, κ-carrageenan, and guar gum, are commonly used in the food industry to improve the rheological properties of starch-based systems and are promising candidates for optimizing 3DFP performance [13,14,15].

While previous studies have examined [16,17] starch-based matrices in 3D food printing applications, few have comprehensively investigated the underlying mechanisms that govern 3D shape accuracy from both a macroscopic (e.g., flow behavior during extrusion) and microscopic (e.g., rheological and mechanical) perspective. Moreover, there is a need to define the critical physicochemical ranges that characterize printable food inks. Therefore, a literature gap emerges for the development of multicomponent material combinations (starches from different botanical sources, protein, and hydrocolloids) as printable food inks.

In this study, we aimed to develop and optimize an extrusion-based 3DFP system and evaluate the printability of composite food inks formulated with different starches (corn and potato), protein concentrates (pea, rice, whey, fava, soy), and hydrocolloids (xanthan gum, arabic gum, κ-carrageenan, carboxymethyl cellulose). In contrast to prior studies that explored more limited ingredient combinations and experimental scope, the present work aims to identify the key material properties governing extrusion behavior and 3D shape fidelity through a combination of experimental rheological and textural analyses. CFD simulations were also employed to gain deeper insight into flow dynamics during extrusion and to determine optimal processing parameters—such as volume flow rate, and nozzle geometry—under varying material conditions, thereby enhancing the precision and efficiency of the 3D food printing process. This combined experimental–computational approach offers the potential to provide a deeper understanding of how formulation and flow dynamics could influence printability, which could support more predictive and standardized food ink development.

## 2. Results and Discussion

### 2.1. Rheological Properties

In order to develop starch-based materials suitable for use as food bioinks, a series of rheological experiments were first conducted to characterize their flow behavior and viscoelastic properties. These tests are essential to assess the printability of the formulations and to understand how different components interact to influence the overall rheological characteristics. The formulations investigated in this study consist of starches derived from two botanical sources—corn (CS) and potato (PS)—combined with various protein concentrates, including fava bean (FP), whey (WP), soy (SP), pea (PP), and rice (RP). In addition, several hydrocolloids were incorporated as structuring agents, namely κ-carrageenan (KC), carboxymethyl cellulose (CMC), xanthan gum (XG), and arabic gum (AG), along with water as the continuous phase. Furthermore, CS and PS were also studied individually in two concentrations of 15 and 20% as reference materials. All materials and their respective abbreviations are listed in the Abbreviations section. Each sample is denoted using a combination of the constituent materials. For example, the abbreviation CSWPKC refers to the formulation composed of corn starch (CS), whey protein (WP), and κ-carrageenan (KC). These combinations were systematically evaluated to determine how each component and their interactions affect the rheological characteristics relevant to extrusion-based 3D food printing.

**Figure 1:**
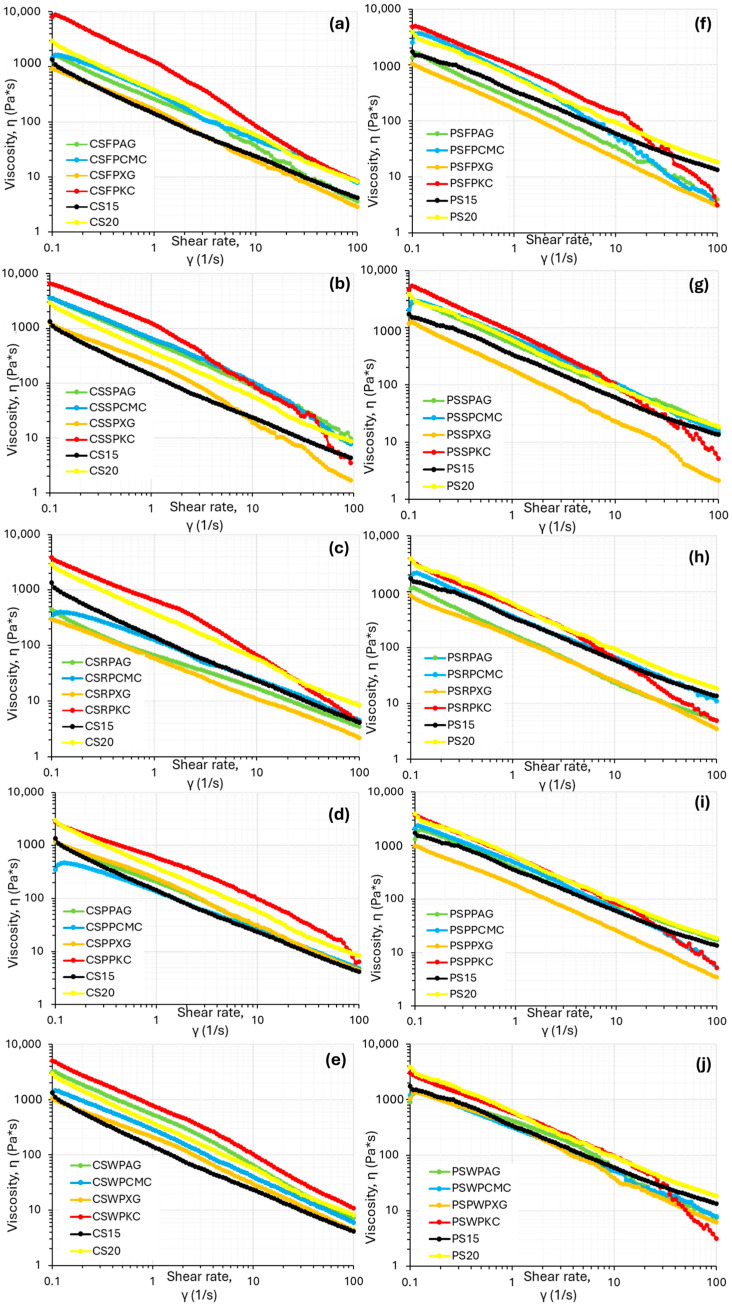
Flow curves of the food ink formulations based on corn starch (**a**–**e**) and potato starch (**f**–**j**) for fava bean protein, soya protein, rice protein, pea protein, and whey protein.

Food inks designed for 3D-printing applications must possess shear-thinning flow characteristics in order to be easily extruded from the syringe outlet, otherwise an increment in friction forces within the syringe barrel could occur, resulting in rough-surfaced, uneven 3D prints [18]. The relationship between viscosity and shear rate is shown in Figure 1, where all studied samples displayed pseudoplastic characteristics (a decrease in viscosity values with the increase in shear rate), which are considered essential for extrusion-based 3D food printing [19]. However, flow behavior was found to be significantly dependent on the hydrocolloid type used.

Control ink formulations (native corn starch without the addition of protein concentrate and hydrocolloid) at the 15% *w*/*v* concentration showed relatively low viscosity values in vicinity of the XG formulations, indicating the promotion effect of hydrocolloids on the viscosity of corn starch due to the gelation of these hydrocolloid molecules. However, the 20% *w*/*v* control ink formulations displayed relatively high viscosity values, but the values were lower than the k-carrageenan formulations (Figure 1). With respect to the control ink formulations based on potato starch, the 15% concentration showed intermediate viscosity values with respect to the different hydrocolloids used, while the 20% concentration displayed increased viscosity values, in cases higher than the KC-based formulations. This phenomenon is possibly related to the observed poor printing quality displayed by this sample.

Starch composite gels with k-carrageenan exhibited the highest viscosity values in comparison with the other hydrocolloid types followed by CMC, arabic gum and xanthan gum, a feature that was evident for both starch types (corn or potato). On the contrary, XG formulations displayed the weakest viscosity profile compared to the other hydrocolloids. Similar results have also been reported in the existing literature, where a decrease in viscosity values has been observed with XG addition in potato starch ink formulations and β-carotene loaded yam-starch-based hydrogel [20,21]. This feature is possibly attributed to the rigid, rod-like structure of XG that aligns more easily under shear compared to a random-coil structure, resulting in an enhanced shear-thinning behavior [22].

Potato starch, as can be seen in Figure 1, also displayed the same pattern with respect to the different hydrocolloid types used. However, overall, potato-starch-based food inks showed ink formulations with lower viscosity values compared to corn-starch-based inks. The observed differences between these two starch types may be attributed to variations in amylose content (potato starch has lower amount) and phospholipid content—potato starch typically contains more phospholipids than corn starch—as well as differences in starch granule morphology, with potato starch having a larger granule size than corn starch [23].

**Figure 2:**
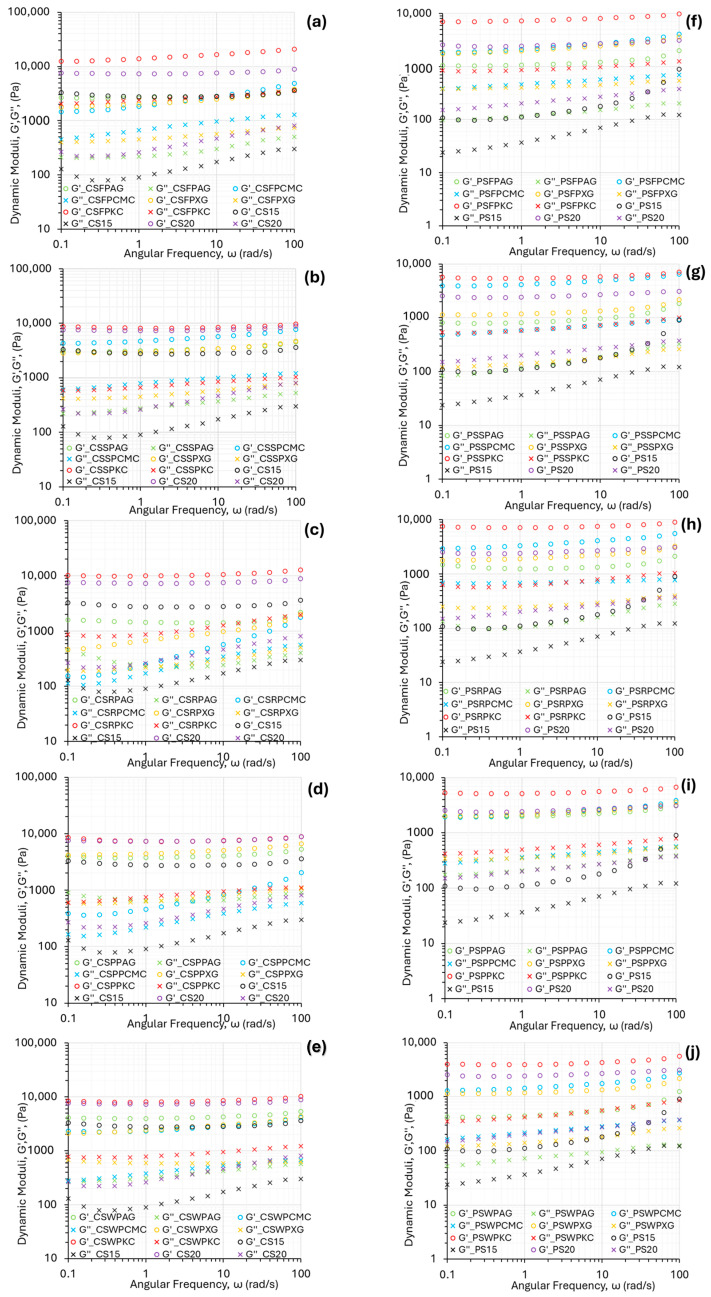
Mechanical spectra of the food ink formulations based on corn starch (**a**–**e**) and potato starch (**f**–**j**) for fava bean protein, soya protein, rice protein, pea protein, and whey protein.

Storage modulus (G′, Pa) is description of the elastic, solid-like behavior of the material and reflects the mechanical strength of the material to withhold deformation. Figure 2 displays the oscillatory frequency sweeps at a constant deformation of all the starch composite gel systems studied. Both dynamic moduli (G′, G″) increased with angular frequency, which is characteristic of materials able to form a strong and stable gel network that can withstand deformation over time [24]. This feature is essential for dimensional stability of 3D printed constructs. The loss modulus (G″, Pa) was significantly lower than G′, indicating the elastic predominance and therefore solid-like behavior that is a desirable feature in the 3D-printing process, suggesting structural integrity of the printed structure [25].

The relationship between the storage and loss moduli (G′ and G″) plays a critical role in determining the suitability of food inks for 3D printing. These materials must withstand the shear forces encountered during extrusion while also being able to recover their shape after deposition. Striking the right balance between G′ and G″ is vital, as it influences both the flow behavior under stress and the structural stability after printing—key factors for ensuring precision and high resolution in printed designs [26].

In the same context, with viscosity data, k-carrageenan formulations, followed by native corn starch slurry at the 20% *w*/*v* concentration, displayed the highest magnitude in G′ and G″ values, while the lowest values for the dynamic moduli were presented by native corn starch slurry at the 15% *w*/*v* concentration. This behavior reveals a higher entanglement degree of the molecular chains of the biopolymers involved, which make the food ink network structure more solid. In the case of ink formulations based on rice protein concentrate, the addition of hydrocolloids, with the exception of KC, was not shown to improve the elastic nor the viscous response of the starch-composite gel system.

The mechanical spectra of potato-starch-based ink formulations, as shown in Figure 2f–j, exhibited the same tendencies, with the lowest values of both moduli being observed for native potato starch food inks with the 15% *w*/*v* concentration, and the highest values for composite gels with KC for all studied protein types. A significantly lower magnitude in both moduli was established in comparison with the corn-starch-based food inks.

Dynamic loss tangent (tan δ = G″/G′) values were compared between different food ink formulations, as shown in Table 1. Materials possessing a low tan δ valued suggest a more prominent solid-like character, while high tan δ values portray materials with a more fluid-like character [27]. For all studied food inks, tan δ values were less than unity, which is characteristic of food with typical weak gel rheological properties. As it has been established in previous research, materials possessing low tan δ exhibited a solid state with poor fluidity, with the tendency to lead to poor extrudability and fracture [20].

The tan δ of samples with the addition of KC displayed decreased tan δ values with respect to the other hydrocolloid types for both starch types, suggesting a higher elastic property of these samples. This feature could further contribute in increasing 3D-printing materials structural integrity post-printing. However, lower tan δ values established for samples such as CS20 and CSFPAG (tan δ < 0.1), displayed poor fluidity, which was indicative of the materials’ flow difficulty in extrusion through the nozzle tip. This could explain the difficulty experienced during printing samples, and their failure to form a complete cuboid structure. Similar low tan δ values have been previously reported in the literature for mashed potatoes with added hydrocolloids [20]. Overall, the results suggest that both the type of hydrocolloid and protein concentrate are critical to the flow behavior and mechanical solid property of the gel samples. Thus, tan δ emerges as a critical predictor of printing performance, bridging rheological measurements with actual structural fidelity.

For a successful 3D-printing process a sufficient level of mechanical strength in the material is a prerequisite to withstand the successive layers of the deposited material. This material ability is reflected by the yield stress (τ_y_) value, descriptive of the minimum stress required for fluid flow and suggestive of the ink’s extrusion ability. A reduction in the τ_y_ values indicates enhanced extrudability for the material, whereas higher τ_y_ values better support performance [28]. In Table 1, starch composite gels prepared with k-carrageenan displayed a significant increase in their τ_y_ values, augmenting the material’s mechanical strength. This feature for KC-formulated inks, as it was established from the printability determinations, facilitated the fabrication of highly accurate 3D-printed constructs with high resistance in deformation. Materials with τ_y_ values ranging from 1269 to 4392 Pa, showed good printability features, while lower τ_y_ values suggested poor printability.

Across all the tested formulations, the best printing performance was consistently associated with a specific rheological profile. These high-performing inks exhibited storage modulus (G′) values exceeding 4000 Pa, loss modulus (G″) values in the range of 500–1100 Pa, and yield stress (τ_y_) above 120 Pa. The tan δ values fell between 0.096 and 0.169, indicating a predominance of elastic behavior while retaining sufficient viscous flow to support extrusion. Notably, inks based on corn or potato starch in combination with κ-carrageenan and protein concentrates such as fava, whey, or rice displayed this favorable rheological window, correlating with excellent shape fidelity and mechanical strength in the printed structures.

The Ostwald-de Waele power-law model (Equation (1)) was employed to describe food inks pseudoplasticity through changes in the consistency coefficient k (Pa*s^n^) and flow behavior index (n values) (Table 2). The correlation coefficient (R^2^) was used as the indicator for the fit of the model. Over the tested shear-rate range, all the tested food-ink systems displayed power-law liquid characteristics with high R^2^ values (0.96–1.00). The k values of the starch composite gels with k-carrageenan were significantly greater than the other hydrocolloid agents, describing the greater thickening ability of k-carrageenan over the other studied types. The use of polysaccharides, such as κ-carrageenan has been highlighted in the literature as a functional means for enhancing the mechanical properties and increasing the viscosity of food inks [29,30]. Therefore, it can be speculated starch composite gels enhanced with appropriate hydrocolloids and proteins could be more effectively applied to 3D printing.

### 2.2. Thermal Properties

The control in the degree of starch gelatinization by heating to different temperatures has been shown to impact the printing quality of starch-based edible inks [20]. The thermodynamic parameters of the gelatinization of native starch and starch-composite gels determined by differential scanning calorimetry (DSC) are depicted in Table 3 and Table 4. For corn starch formulations no significant differences were observed with respect to T_on_ and T_p_, while a slight decrease in T_end_ was observed in comparison with native corn starch. The decrease in gelatinization enthalpy values was evident for both starch types in comparison with the native starch samples. This phenomenon is most likely attributed to the competitive interactions among the starch, hydrocolloid, and protein for the available water in crystalline regions that ultimately results in the partial gelatinization of these regions [31].

### 2.3. Texture Properties

**Figure 3:**
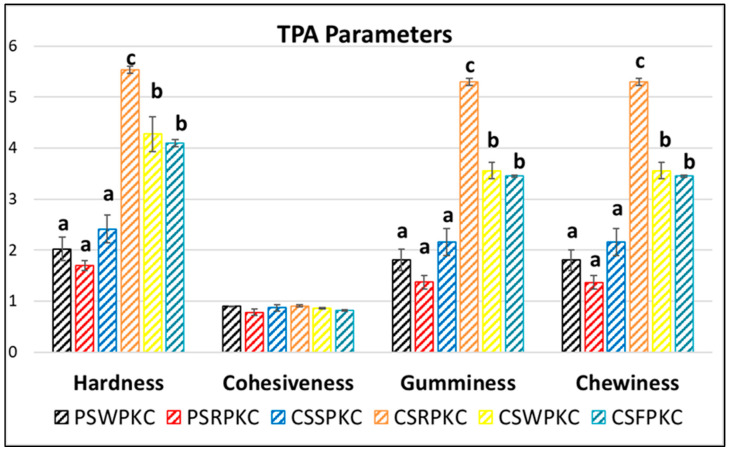
Texture Profile Analysis parameters of the food inks. Bars sharing different letters within the same textural attribute are significantly different at *p* < 0.05.

TPA (Texture Profile Analysis) tests can be performed to evaluate the textural attributes of 3D-printed food, simulating its oral perception. The mechanical profile not only affects mouthfeel but also determines the stacking behavior during printing. Hardness and chewiness are particularly critical for vertical buildability and stability. Figure 3 displays the texture profiles of the 3D-printed structures with the best printing performance, as shown in Section 2.5. The parameter of hardness describes the required force for deformation of foods. Specifically, the corn-starch-based food inks were found to possess significantly higher hardness values in comparison to the potato-starch-based food ink formulations.

The cohesiveness value is related to the energy required to disrupt the internal structure of a gel [32]. Regarding cohesiveness, which is indicative of the adhesion amongst the deposited subsequent layers, no impact (positive or negative) was found with respect to food ink formulation. The parameter of gumminess, representing the amount of required energy to fracture a sample’s semi-solid structure to a swallowing state, showed the same trend, with the corn-starch-based formulations exhibiting increased values compared to the potato-starch-based formulations. Chewiness, displaying the mastication properties of the 3D-printed structures, also exhibited the same tendency. Increased gumminess and chewiness values could be beneficial for chewy snack food, since the mouthfeel of the gels in the tongue, teeth, or palate is often desirable. However, this textural attribute may also pose risks for safe swallowing [33].

Overall, it is essential to develop food products tailored to diverse functional needs, such as those required by individuals with swallowing difficulties (dysphagia). Dysphagia appears in varying degrees of severity, each requiring specific textural modifications to ensure safe and acceptable consumption. In this context, 3D-printing technology has been utilized as a promising approach for designing foods with controlled internal structures and well-defined textural properties, as reported in previous studies [34,35,36]. Based on the texture analysis of the food inks developed in this study, a classification system could possibly be established for the categorization of inks according to their suitability for different levels of dysphagia. This classification would be based on the measured hardness values and the specific raw materials used, including the botanical origin of the starch and the type of protein concentrate.

### 2.4. Color Properties

**Figure 4:**
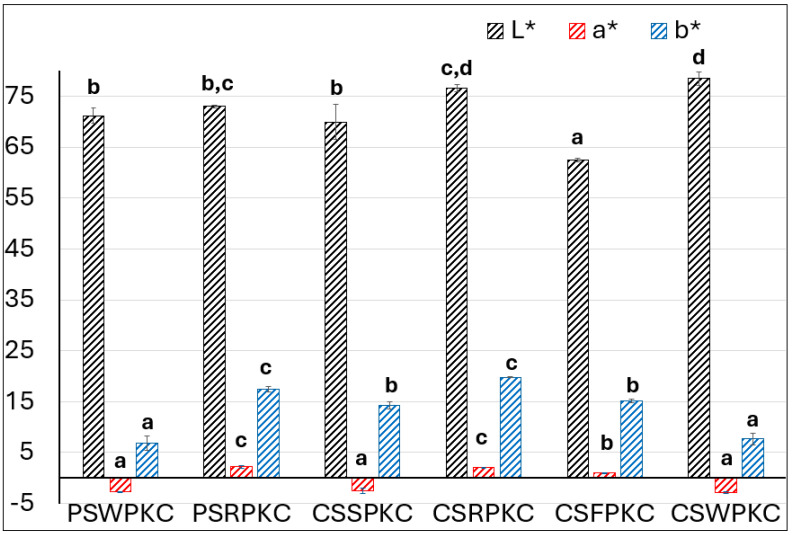
Chromatic parameters evaluation of the food inks. Bars sharing different letters within the same chromatic parameter are significantly different at *p* < 0.05.

The color properties of the food inks were determined in the CIELab space and are depicted in Figure 4. The lightness (L) values were lowest for the corn-starch-based formulations containing fava bean protein concentrate and highest for those prepared with whey protein. The observed differences are attributed to the inherent colors of the protein concentrates, with whey protein exhibiting a white, milky appearance and fava bean protein displaying a more yellow hue.

The parameter of a* corresponds to the redness of the samples, where negative values were displayed for PSWPKC, CSSPKC, and CSWPKC. This can also be attributed to the protein source that was added into the food ink formulations, with the whey protein and rice protein concentrate exhibiting a more green intensity.

With respect to the parameter of b*, the samples prepared with rice protein concentrate in both starch types displayed the highest values, indicating the yellow intensity of the food inks. Overall, the observed differences in the evaluated color parameters are primarily related to the color hue of the protein concentrate source used.

### 2.5. Printing Accuracy Determination of the Food Inks

The first screening step for the evaluation of the printing ability of all the tested food inks was performed as displayed in Tables S1 and S2. A print score of 0–5 (0—Unprintable and 5—Good Printability) was used as a first printability indicator, based on visual observations of 3D prints regarding distinction between subsequent layers, extrusion difficulty, continuous or intermittent printing, merged layers, and self-support ability or underweight spreading.

**Figure 5:**
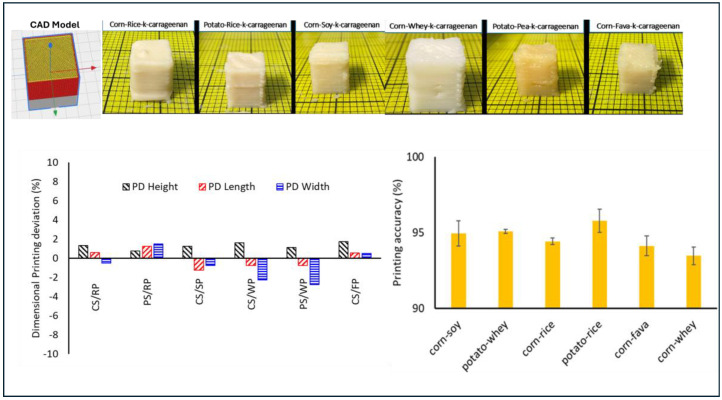
Dimensional printing deviation (%) and printing accuracy evaluation of food inks.

The prepared food inks with the highest printing score (5—Good Printability) were further investigated in terms of % dimensional printing deviations and the printing accuracies of the 3D-printed products were investigated. Figure 5 displays the visual observations of the 3D-printed objects with the best printing characteristics.

Food ink formulations with optimal printing characteristics (CSRPKC, CSSPKC, CSFPKC, CSWPKC, PSRPKC, PSWPKC) displayed a unique balance that allowed both smooth extrusion through the nozzle and structure rigidity to prevent deformation after deposition. These formulations were characterized by a viscosity profile enabling a smooth extrusion through the nozzle and quick solidification after deposition, with self-supporting properties and shape fidelity.

The dimensional printing deviations that refer to variations in diameter and height dimension from the 3D CAD model are displayed in Figure 5. All six cuboid structures displayed were successfully printed. The lowest dimensional printing deviation, which represents the highest printing accuracy, was achieved for the PSWPKC, CSWPKC, and CSSPKC samples, with 0.75% and 2.75%, 0.75% and 2.25%, and 1.25% and 0.75% for length and width, respectively. All samples displayed positive values for height, corresponding to the largest printing deviation from the CAD model, which suggests a higher structure than the designed model. This is translated in the creation of a thicker than desired outline in the cuboid structure. Among the samples studied, PSRPKC, CSFPKC, and CSRPKC displayed the highest printing deviations, which were exhibited as inconsistencies in length and width and low smoothness in printed lines amongst the deposited printed layers. This phenomenon could be primarily attributed to the viscosity profile of the selected food inks. CSFPKC displayed the highest viscosity and gel strength compared to the other formulations studied, which could cause increased friction level within the syringe barrel, and thus difficulty during extrusion [37]. In this context, PSRPKC and CSRPKC displayed the lowest viscosity values among the studied systems, which resulted in a higher material spreading.

Regarding the printing accuracy of the printed structures, a high accuracy level was established for all the studied samples (approximately 95%) with the highest score measured for PSRPKC (95.8%) and the lowest for CSWPKC (93.8%). Interestingly, minor variations in hydrocolloid–protein combinations translated to marked differences in structural outcomes, emphasizing the sensitivity of printability to formulation fine-tuning.

### 2.6. Flow Simulation of the Food Inks

**Figure 6:**
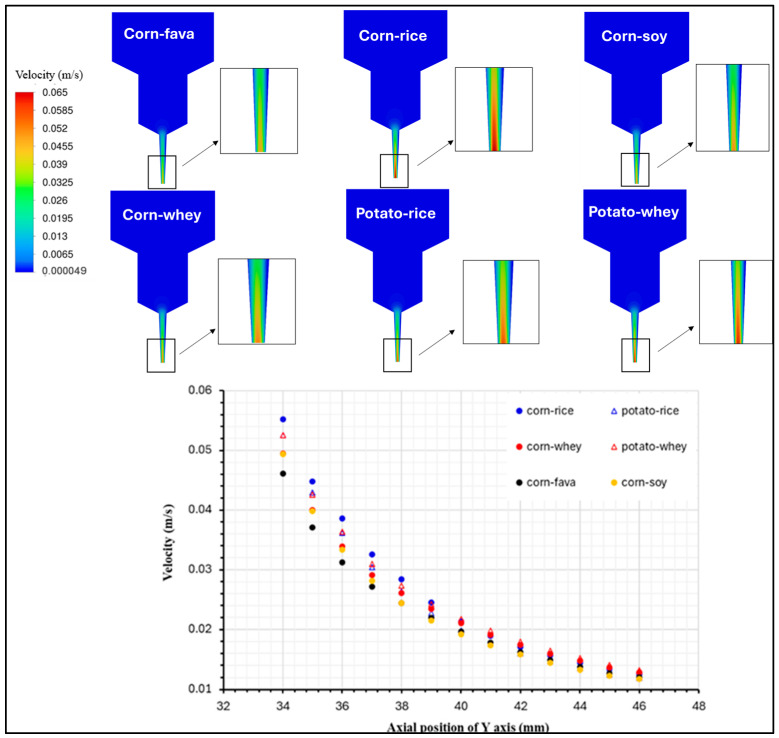
Velocity distribution profile for food ink formulations with the best printability.

Figure 6 illustrates the simulated velocity distribution within the flow field, obtained via CFD, for the food inks with the best printing performance, which exhibit differences in their viscosity profiles. It has been demonstrated that a uniform extrusion pattern, in terms of speed at the nozzle’s outlet and the material’s post-printing shape accuracy is directly related to the velocity field inside the extrusion syringe system [38]. This simulation of velocity distribution is indicative of the material’s flow within the extrusion syringe, which is dependent on its rheological properties. The highest velocity values can be observed close to the nozzle’s outlet, where the syringe’s geometry narrows, while the lowest values are located in the beginning of the flow field.

The velocity profiles along the y-axis displayed differences for the six different samples, where the velocity magnitude was significantly affected by the viscosity profile of each sample. The highest velocity corresponded to an enhanced fluidity of the sample through the syringe nozzle, when pressure was applied through the syringe piston. On the contrary, the lowest velocity profiles were exhibited for the food inks with the highest viscosity values, namely CSFPKC and CSSPKC. This feature is characteristic of the difficulty level that the food ink is experiencing during its extrusion. The velocity profiles described are in accordance with findings of starch composite gels [39]. The illustration of the velocity distribution can enhance the comprehension of the material’s flow behavior, which can further contribute to the development and prediction of the final product’s quality attributes.

**Figure 7:**
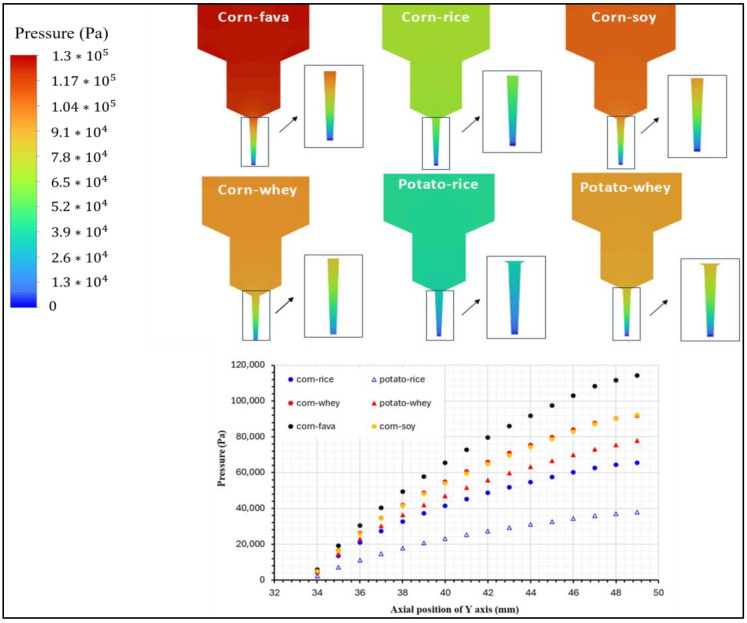
Pressure distribution profile for the food ink formulations with best printability.

The distribution of pressure inside the 3DFP syringe system is illustrated in Figure 7. For a successful uniform and continuous extrusion of a food ink 3DP process, the paste in the chamber is pushed through the die, i.e., the end part of the nozzle, by applying a pressure force relative to the volume flow rate. Printing materials used for 3DFP processes must receive an appropriate level of pressure for their continuously smooth extrusion. As shown in Figure 7, pressure distributions of the food inks within the syringe exhibited different pressure magnitudes that were consistent with their viscosity profiles.

As the inks moved through the syringe system, a lower amount of pressure was applied at the ink at the front, while the ink at the back was consistently compressed by the piston’s force. As a result, an increment in pressure was detected in the upper section, requiring higher inlet pressure for inks with greater viscosity. The pressure values on the centerline of the extrusion syringe system were compared for the six food inks. Overall, the corn-starch-based food inks, except for CSRPKC, exhibited higher pressure at the same location compared to the potato-starch-based inks, with CSFPKC and CSSPKC showing the highest-pressure values and PSWPKC the lowest generated pressure. This feature is consistent with the rheological parameters of the tested food inks, where indeed an increment in viscosity, storage and loss moduli, and yield stress values was observed over the studied inks. In all cases, a gradient decrease in pressure was observed from the inlet to the syringe outlet (nozzle tip), which is suggestive of the required piston pressure, reflecting difficulties in extrudability. The pressure model revealed localized areas of sharply increased pressure near the nozzle. Such regions may lead to flow discontinuities or even clogging during experiments. The pressure drops increased for the corn-starch-based formulations prepared with fava and soy protein concentrates.

Overall, the generated simulations were comparable with the experimental results and provided valuable insights through visualization of the flow dynamics within the syringe, having complementary action on the experimental data limitations. Materials with different viscosities displayed different a velocity distribution within the printer and good fluidity during flow-out. Although materials possessing higher viscosity displayed increased pressure drops between the inlet and the outlet, optimal printing was not prevented, suggesting a printable range for starch-based materials with different viscosities. Consequently, higher piston pressure was required. Furthermore, for the shear-thinning materials studied, we noted that viscosities had similar values at the nozzles even though there were tremendous differences in viscosities at the beginning. This compatibility of rheological data with flow simulation underscores the importance of pre-print rheological screening as a predictor of performance under simulated extrusion stress.

Regarding the shear-stress distribution simulation of flow through the nozzle, no significant differences were established among different food ink formulations studied (Figure S5).

### 2.7. FTIR of the Food Inks

**Figure 8:**
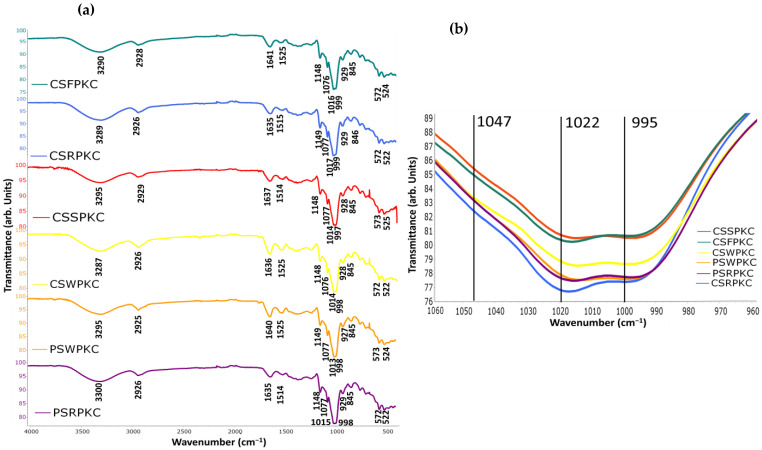
FTIR spectra of the food inks. (**a**) Full spectra in the range 4000–400 cm^−1^. (**b**) Magnified view of the spectral region from 1060 to 960 cm^−1^ for all food inks.

The short-range ordered structure and functional group changes of the starch-based gels were analyzed by infrared spectroscopy (Figure 8). In the wavelength range between 4000 and 400 cm^−1^, no new functional groups were observed in any of the samples, indicating that the addition of the hydrocolloids did not lead to the formation of any new covalent bonds. Overall, similar spectra were obtained for the different biopolymer food inks, with only slight differences in transmittance values. Moreover, when compared with the individual spectra of each component (starches, hydrocolloids, and proteins), the composite spectra exhibited decreased transmittance peaks with slight shifts, while no new peaks appeared. This is in agreement with previous studies, which reported that during gelatinization with hydrocolloid addition, natural interactions between components can be observed rather than changes in the chemical structure or the creation of new functional groups [21].

The broad absorption peak observed in the range between 3700 and 3000 cm^−1^ is typically associated with the molecular bond -OH tensile vibrations, which reflects the formation of hydrogen bonds between the different components [40]. Hydrogen bond formation is suggestive of protein and polysaccharide interaction, which can enhance cross-linking strength and thus improve the strength of the formed network structure, which is an essential factor governing the printability of biomacromolecule-based food inks [41]. Peaks were also identified at approximately 2925 and 1148 cm^−1^, which can be attributed to the tensile and bending vibrations of the C-H bands in the starch and hydrocolloids. The peak observed near the absorption region of 1640 cm^−1^ is associated with the O-H bending vibration of the water molecules in the sample [42]. Since no new chemical bonds were formed with the addition of protein concentrate and hydrocolloids in the starch matrix, the increase in gel strength in the starch composite gels is possibly linked with the hydrogen bonding and electrostatic interactions among the components. In the studied biopolymer mixtures, protein-related bands corresponding to Amides I and II (1641–1634, 1525–1514, and 524–522 cm^−1^) were also observed. These bands exhibited reduced intensity, possibly due to the lower concentration of protein relative to starch.

Bands at 995 cm^−1^ could be used as indicators of the variations in hydrated starch structure, while bands at 1022 cm^−1^ have been known to display sensitivity to amorphous and short-range ordered starch structures [43]. The 995/1022 cm^−1^ ratio has been recognized as a reliable indicator of the degree of double helical structure in starch. Specifically, a lower ratio suggests a higher proportion of amorphous starch relative to its crystalline counterpart. Among the samples analyzed, only minor variations were observed: formulations containing whey and soy proteins (PSWPKC, CSSPKC, CSWPKC) exhibited higher ratios, indicating a greater degree of crystallinity, while those with rice and fava proteins (CSRPKC, PSRPKC, CSFPKC) showed comparatively lower values, suggesting a higher amorphous content. In a previous study by Jiao et al., it was demonstrated that a high degree of gelatinization may be unfavorable for extrusion in 3D-printing applications [44].

Another useful spectral ratio for evaluating the crystalline structure and the extent of short-range molecular ordering in starch is the 1047/1022 cm^−1^ ratio. The absorbance at 1047 cm^−1^ is also associated with starch crystallinity [45]. Compared with pure starch samples, the degree of order of all the samples decreased. This effect is possibly attributed to the interaction between the hydrocolloids and starch that caused a reduction in the crosslinking between starch molecules, thereby partially disrupting their short-range order [46]. This finding has been reported in similar research where xanthan gum addition to chestnut starch resulted in a decrease in the degree of order of the starch [47]. Based on the obtained spectra, a similar trend was observed as with the 995/1022 ratio, with the PSWPKC, CSSPKC, and CSWPKC samples exhibiting higher values.

## 3. Conclusions

In conclusion, this study presents a detailed evaluation of various starch-based bioink formulations incorporating different proteins and hydrocolloids. Our findings indicate that the composition and interactions among starch, protein, and hydrocolloid components play a key role in determining the rheological behavior, mechanical stability, and overall printability of the gels. The results highlight the potential of optimizing ingredient combinations to develop tailored bioinks with desirable performance characteristics for extrusion-based applications in the food sector. Among the formulations tested, starch-based gels incorporating κ-carrageenan (KC) consistently exhibited superior rheological behavior, higher mechanical strength, and enhanced printability. Specifically, food inks combining corn or potato starch with fava, whey, rice, or soya proteins and κ-carrageenan achieved the highest storage modulus (G′), appropriate yield stress (τ_y_), and excellent structural fidelity (93–96% printing accuracy). Rheological analyses revealed that successful extrusion and post-deposition shape retention were associated with tan δ values between 0.096–0.169 and G′ values exceeding 4000 Pa. Corn-starch-based inks showed superior textural attributes (e.g., hardness, chewiness), making them potentially better suited for applications requiring firmer textures, such as customizable snacks. In parallel, potato-starch-based formulations displayed decreased values in texture parameters, highlighting their potential use as food inks for specific consumer needs (e.g., dysphagia). These findings are insightful for the formulation of 3DFP-compatible edible gels with tailored mechanical and sensory properties. Computational Fluid Dynamics (CFD) simulations supported experimental observations, confirming that pressure and velocity distributions within the syringe correlated with the inks’ consistency index (k). FTIR analysis suggested that improvements in gel strength were due to non-covalent interactions, particularly hydrogen bonding and electrostatic effects, rather than chemical modifications. This technology allows for precise control of food texture, shape, and nutritional content, thereby enabling the optimization of printable formulations suitable for varying degrees of dysphagia. Future research should explore the nutritional bioavailability, scaling of 3D-printed foods, and consumer acceptance, which are critical for advancing personalized nutrition and sustainable food manufacturing.

## 4. Materials and Methods

### 4.1. Materials

Potato, corn starch, xanthan gum, arabic gum, and k-carrageenan were purchased from Sigma-Aldrich (Sigma-Aldrich-MERCK, St. Louis, MO, USA). Pea protein (80% *w*/*w* protein dry basis), fava bean protein (55% *w*/*w* protein dry basis), and rice protein (80% *w*/*w* protein dry basis) concentrates were kindly supplied from Kirpitsas ingredients (Kirpitsas Natural Products P.C., Serres, Greece). Soya protein isolate (90% *w*/*w* protein dry basis), whey protein concentrate (80% *w*/*w* protein dry basis), and carboxymethyl cellulose were purchased from Falcon Ingredients (Falcon S.A, Athens, Greece).

### 4.2. Food-Ink Preparation

Solutions of 1% hydrocolloid (*w*/*v*) were prepared by dispersion in deionized water, until complete dissolution. A thoroughly dry-mixed blend of 5% protein concentrate and 15% starch was added in the solution and was agitated for approximately 30 min until a homogenized biopolymer solution was obtained. Control samples without the addition of hydrocolloid and protein were also prepared for both starch types in two different concentrations of 15 and 20% *w*/*v*.

The prepared biopolymer solution sample was then placed in an RCT Basic S1 Digital Hot Plate Magnetic Stirrer (IKA^®^-Werke GmbH and Co. KG, Staufen, Germany) and heated at a constant temperature (80 °C) under agitation until gelatinization (10–14 min depending on the sample). The different food ink compositions and their abbreviations are shown in Tables S2 and S3.

### 4.3. Three-Dimensional Printer Customization

**Figure 9:**
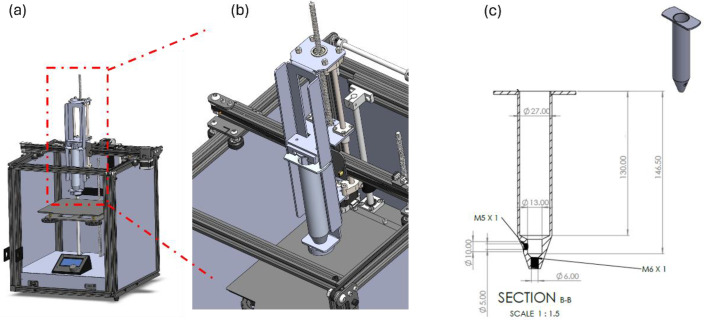
Overview of the extrusion 3D-printing equipment manufactured for the purpose of this study. (**a**) front view of custom-made extrusion 3D printer setup, (**b**) magnified illustration of the extrusion syringe system, (**c**) dimensional design of the printing syringe.

A custom-built extrusion-based 3D printer system based on the working technologies of Computer Numerical Control (CNC) machines with moving parts in three (3) axes (X–Y–Z) was designed for the purpose of this study (Figure 9). For better flow control and capability, a change on main board electric current management was developed so as to enhance the performance of the extruder’s motor. On the printing platform, which allows a building volume of 170 mm in all dimensions, a food-safe, removable and washable silicon matte was placed in order to enhance the traction between the extruded material and the platform and to protect the printed food and preserve the general hygienic operational conditions of the machine. The extrusion head, which is the main part of the printer and also the part where the development focused on, is based on a conventional syringe extrusion system. The barrel’s volume was selected after a thorough study on the flow analysis according to commercially available plungers and the ability to maintain a fine flow during the whole 3D-printing process. The final system design included the ability to extrude up to 60 mL of material in one shot and with a deviation in flow of less than 2% (pressure in the barrel is not the same from the max capability to empty). The syringe needle was also selected from commercially available extrusion nozzles of 3D printers with the ability to interchange nozzles with varying diameters (0.5 mm to 2.0 mm). On the printing head a special designed heating unit was also attached, that can achieve temperatures up to 180 °C. A strong subframe was designed and manufactured for the syringe. The meticulous design enables the ability of quickly removing and placing the syringe on the head, while no compromising exists on the stability of the syringe due to high forces and torques acting on the syringe during extrusion that could create unstable flow of the material. Finally external plastic protection walls were placed. The main specifications described above including some more are below shown in Table S1.

### 4.4. Optimization of the 3D-Printing Conditions

Three dimensional printing of the developed bio-inks was performed using the UltiMaker Cura v5.8 software (Ultimaker B.V., Watermolenweg 2, 4191 PN, Geldermalsen, The Netherlands), which allows the conversion of a 3D designed object into a Gcode, which is then read and printed by the 3D food printer. This software enables the adjustment and control of the basic 3D-printing parameters during the process, as well as additional parameters such as temperature and air flow. Furthermore, the program can virtually slice the printing object into layers, enabling the monitoring of the internal structure of the 3D object. The following print settings were attuned during preliminary experiments: 1 mm nozzle diameter, 1 mm layer height, 100% infill density, grid infill pattern, 20 mm/s print and infill speed, 10 mm/s wall speed, 0.8 mm initial layer height, 1 mm line width, and 2.2% flow rate.

### 4.5. Modelling of the Flow Behavior, Assumptions, and Boundary Conditions

A CFD (Computational Fluid Dynamics) model was established to investigate the flow fields of the six food inks with the best printing performance, as determined in Section 2.5, in the extrusion syringe of the custom 3D-printing setup with the Ansys^®^ Academic Research software (23.2). The prepared biopolymer inks were considered as isothermal, incompressible, high-viscosity fluids with laminar flow and non-Newtonian behavior. Due to inks high viscosity values, the forces of inertia and gravity were omitted. Experimental data were fitted to the power-law model, defining the relationship between apparent viscosity and shear rate according to Equation (1):

(1) $$\eta =k*\gamma^{n-1}$$

where η is the viscosity (Pa), k is the consistency index (Pa*s^n^), n is the flow behavior index, and γ is the shear rate.

The 3D geometry of the cylindrical syringe and nozzle was reconstructed in CAD (ANSYS^®^ Academic Research Design Modeler, Release 23.2) based on precise laboratory measurements. The geometry was refined near the nozzle region to accurately capture local gradients in pressure and velocity. Three boundary faces were defined: inlet, outlet, and walls.

Mesh generation was performed automatically with an average element size of 0.024 mm, ensuring sufficient resolution in the nozzle region, where flow gradients are most pronounced. Numerical simulations were conducted in ANSYS^®^ Academic Research Fluent, Release 23.2 using the Finite Element Method (FEM) under steady-state and isothermal conditions.

Boundary conditions consisted of a velocity inlet at the top surface to represent piston-driven flow and a pressure outlet at the nozzle tip. All wall surfaces were assigned no-slip conditions. The simulation was considered converged when residuals dropped below 10^−6^.

### 4.6. Rheological Properties of the Food Ink

Rheological characterization of the materials was performed on a stress-controlled rotational rheometer (MCR 102, Anton Paar, Graaz, Austria) using a parallel plate geometry (PP25). The magnitudes of storage modulus (G′) and loss modulus (G″), demonstrating the elastic and viscous properties of the material, alongside the phase angle δ (tan δ, G″/G′, representing the phase difference between stress and strain during oscillation) were recorded and analyzed as a function of angular frequency (ω, 0.1–100 rad/s) with the Anton Paar RheoCompass Analysis Software (Version 1.15.445, Graz, Austria). All measurements were performed in triplicate.

#### 4.6.1. Amplitude Sweep

Oscillatory amplitude sweep measurements were performed for the determination of the linear viscoelastic region (L.V.R.), in the range of 0.01–100% and a frequency of 1 Hz at ambient temperature (25 °C). Yield stress (τ_y_), corresponding to the stress value at G′ = G″, was determined.

#### 4.6.2. Frequency Sweep

Frequency sweep (at the strain of 0.1%, within the L.V.R.) measurements were performed in the range of 0.1–100 rad/s angular frequency at ambient temperature (25 °C).

### 4.7. Thermal Properties of the Food Inks

The thermal properties of biopolymer blends were determined using a Differential Scanning Calorimeter (DSC 6000, Perkin Elmer, Waltham, MA, USA). The samples (dry biopolymer blends) were weighed and hydrated directly into the aluminum pans (5 g dry blend/15 mL deionized water) and were subsequently hermetically sealed. An empty aluminum pan was used as a reference and at least three runs for each sample were performed. The DSC temperature program consisted of a temperature scan from 30 to 120 °C at a scan rate of 10 °C/min. The onset temperature (T_o_), the peak temperature (T_p_), the endset temperature (T_end_), and the enthalpy (ΔH) associated with the starch gelatinization were calculated with the aid of the Pyris software (V. 9.1).

### 4.8. Printability Accuracy Determination

For the determination of printability accuracy, two different evaluation methods were employed. The method of dimensional printing deviation was performed, according to Liu et al., (2018) method [20]. A cuboid shape (length 20 mm, width 20 mm, and height 20 mm) was designed with UltiMaker Cura v5.8. (Ultimaker B.V., Watermolenweg 2, 4191 PN, Geldermalsen, The Netherlands). The three dimensions (length, width, height) of each printed structure (at least three replications for each sample) were measured by means of a Vernier caliper. The magnitude of dimensional printing deviation was determined according to Equation (2):

(2) $$\mathrm{Printing}\;\mathrm{deviation}\;\left(\%\right)=\frac{\left(\mathrm{Measured}\;\mathrm{dimentions}\;\mathrm{value}-\mathrm{Target}\;\mathrm{dimensions}\;\mathrm{value}\right)}{\mathrm{Target}\;\mathrm{dimensions}\;\mathrm{value}}*100$$

The magnitude of printing deviation can dictate the printing accuracy of the 3D-printed constructs. Positive values indicate poor printing accuracy properties, where printed objects possess thicker outlines than desired. Respectively, negative values indicate thinner outlines for the printed structures, thus, more proximate to the target dimensions. The 3D-printed structures with the best printing deviation (%) performance were selected for the further determination of their physicochemical properties.

An additional method for the evaluation of printing accuracy was also employed according to Equation (3), as described by Xu et al. (2023) [48]:

(3) $$\mathrm{Printing}\;\mathrm{accuracy}\;\left(\%\right)=\;\frac{\left(1-\frac{H_{S}-H_{E}}{H_{E}}\right)+\left(1-\frac{H_{S}\;-{\;H}_{C}}{H_{S}}\right)+\left(1-\frac{L_{S}-{\;L}_{b}}{H_{S}}\right)}{3}*100$$

where H_e_ corresponds to the edge height (mm), H_c_ to the center height (mm), and H_s_ to the set height (mm) of the 3D-printed structure. L_b_ is defined as the bottom length (mm) and L_s_ as the set bottom length (mm) of the cuboid model.

### 4.9. Texture Properties of the 3D Prints

The texture properties of 3D prints, namely hardness, chewiness, springiness, and gumminess, were determined using a texture analyzer (TA.XT Plus, Stable Microsystem Ltd., Godalming, UK) equipped with a P/6 cylinder probe. Samples were compressed uniaxially at 40% of their height with a test speed of 100 mm/s and a 10 s interval between compressions [49]. All measurements were conducted at room temperature (25 ± 1 °C) in at least four replications.

### 4.10. Color Properties of the 3D Prints

Color properties of the printed structures were investigated as a function of different food-ink formulas and measured by means of a spectrophotometer (CM-5, Konica Minolta Co., Tokyo, Japan). Prior to each measurement zero (0% calibration, baseline correction) and white calibration (100% calibration, brightest color standard) was performed. The appropriate target mask (CM-A203, Konica Minolta Co., Tokyo, Japan)) was selected for the reflectance measurement by means of petri dish (CM-A128, Konica Minolta Co., Tokyo, Japan). Color parameters described in numerical values of L*, a*, and b* were calculated with the Color Data Software CM-S100w, SpectraMagic NX Lite (Ver.3.31, Konica Milolta, Inc.). The L* parameter describes the lightness of the samples. The a* parameter describes the sample’s redness (positive values point to red intensity and negative values to green), whereas the b* parameter determines the sample’s blueness (positive values display yellowness while negative values describe blueness). Measurements were performed at least in triplicate.

### 4.11. Fourier Transform Infrared Spectroscopy with Attenuated Total Reflectance (FT-IR-ATR) Analysis

FT-IR spectral absorption performance for the prepared food inks was assessed by means of an FT-IR-ATR spectrometer (Spectrum 100, Perkin Elmer, Waltham, MA, USA). The food inks were subjected to freeze drying (Biobase BK-FD10P, Jinan, China) prior to measurements and were ground into a fine powder. Measurements were performed at ambient temperature with 64 scans and are displayed as a mean of three replications. The resulting spectra were obtained within a wavenumber range of 4000 cm^−1^ to 400 cm^−1^ with a scanning resolution of 1 cm^−1^. Background effects were removed by performing a spectrum of the empty cell. Spectraglyph software (V 1.2.16.1) was used for spectra analyses.

### 4.12. Statistical Analysis

The results were analyzed using a statistical software program SPSS 21.0 software package (SPSS Inc., Chicago, IL, USA). One-way ANOVA and post-hoc Tukey test were used to determine statistically significant differences between different food inks samples, with respect to each starch type (*p* < 0.05).

## Figures and Tables

**Table 1:** Viscoelastic parameters for all the food ink formulations at frequency of 1 Hz.

Samples	Storage Modulus, G′, (Pa)	Loss Modulus, G′, (Pa)	tan δ (Dimensionless)	Yield Stress, τ_y_, (Pa)
CSFPAG	2917 ± 83 ^b,c,d,e,f^	285 ± 12 ^a,b^	0.098 ± 0.002 ^b,c^	392 ± 15 ^b^
CSFPCMC	2637 ± 78 ^a,b.c,d^	912 ± 13 ^e,f^	0.346 ± 0.006 ^h^	517 ± 13 ^b^
CSFPKC	15,553 ± 196 ^k^	2623 ± 86 ^g^	0.169 ± 0.001 ^e,f^	3032 ± 54 ^f^
CSFPXG	2316 ± 61 ^a,b,c,d,e^	536 ± 7 ^b,c,d^	0.232 ± 0.003 ^g^	349 ± 8 ^b^
CSRPAG	1551 ± 92 ^a,b,c,d^	236 ± 15 ^a,b^	0.158 ± 0.021 ^d,e,f^	257 ± 10 ^a^
CSRPCMC	446 ± 36 ^a^	282 ± 11 ^a,b^	0.633 ± 0.021 ^j^	181 ± 4 ^a^
CSRPKC	9707 ± 110 ^j^	1114 ± 7 ^f^	0.115 ± 0.004 ^c,d^	2652 ± 46 ^e,f^
CSRPXG	974 ± 13 ^a,b,c^	311 ± 4 ^a,b,c^	0.319 ± 0.003 ^h^	361 ± 16 ^a^
CSSPAG	3234 ± 93 ^e,f,g^	3489 ± 72 ^a,b,c^	0.108 ± 0.006 ^c^	1579 ± 26 ^c,d^
CSSPCMC	5231 ± 98 ^g,h^	908 ± 18 ^f^	0.181 ± 0.008 ^f,g^	1497 ± 31 ^c,d^
CSSPKC	8174 ± 101 ^i,j^	791 ± 8 ^d,e,f^	0.097 ± 0.000 ^a,b,c^	4392 ± 80 ^g^
CSSPXG	3348 ± 32 ^d,e,f,g^	548 ± 13 ^b,c,d^	0.165 ± 0.003 ^e,f^	757 ± 16 ^a,b^
CSPPAG	3923 ± 29 ^e,f,g^	626 ± 33 ^c,d,e^	0.160 ± 0.005 ^d,e,f^	1436 ± 21 ^b,c^
CSPPCMC	747 ± 13 ^a,b^	360 ± 13 ^a,b,c^	0.482 ± 0.004 ^i^	228 ± 8 ^a^
CSPPKC	8222 ± 95 ^i,j^	853 ± 15 ^f^	0.139 ± 0.042 ^c,d,e^	4003 ± 9 ^g^
CSPPXG	4905 ± 43 ^f,g^	775 ± 9 ^d,e,f^	0.158 ± 0.004 ^d,e,f^	1748 ± 15 ^c,d^
CSWPAG	4184 ± 22 ^e,f,g^	359 ± 10 ^a,b,c^	0.092 ± 0.005 ^a,b,c^	1719 ± 26 ^c,d^
CSWPCMC	2812 ± 78 ^b,c,d,e^	514 ± 11 ^b,c,d^	0.183 ± 0.002 ^f^	1291 ± 68 ^c^
CSWPKC	8238 ± 95 ^i,j^	854 ± 18 ^d,e,f^	0.104 ± 0.004 ^c^	2192 ± 80 ^d,e^
CSWPXG	2833 ± 71 ^b,c,d,e,f^	566 ± 13 ^b,c,d^	0.200 ± 0.006 ^f,g^	1408 ± 23 ^b,c^
CS15	2998 ± 249 ^b,c,d,e,f^	151 ± 17 ^a^	0.050 ± 0.02 ^a^	123 ± 5 ^a^
CS20	7731 ± 1106 ^i^	419 ± 55 ^b,c,d^	0.054 ± 0.02 ^a^	582 ± 14 ^b^
PSFPAG	1149 ± 85 ^a,b,c,d^	142 ± 7 ^a,b,c^	0.123 ± 0.001 ^a,b,c^	4450 ± 15 ^a,b,c,d^
PSFPCMC	2608 ± 18 ^g^	520 ± 19 ^i^	0.201 ± 0.018 ^h^	984 ± 73 ^e,f^
PSFPKC	7871 ± 104 ^j^	1003 ± 87 ^i^	0.127 ± 0.008 ^a,b,c^	2154 ± 52 ^j^
PSFPXG	2324 ± 29 ^e,f,g^	439 ± 14 ^c,d,e^	0.189 ± 0.004 ^g,h^	505 ± 27 ^b,c,d^
PSRPAG	1375 ± 42 ^b,c,d,e,f^	163 ± 10 ^a,b,c^	0.118 ± 0.004 ^a,b,c^	569 ± 17 ^c,d^
PSRPCMC	3997 ± 64 ^h^	740 ± 31 ^i^	0.188 ± 0.017 ^g,h^	647 ± 16 ^d,e^
PSRPKC	7382 ± 99 ^j^	712 ± 41 ^i^	0.096 ± 0.002 ^a^	1466 ± 55 ^h,i^
PSRPXG	2129 ± 95 ^d,e,f,g^	283 ± 16 ^c,d,e^	0.133 ± 0.001 ^b,c,d^	556 ± 15 ^b,c,d^
PSSPAG	1002 ± 34 ^b,c^	177 ± 14 ^a,b,c^	0.177 ± 0.005 ^b,c,d,e^	666 ± 43 ^d,e^
PSSPCMC	4609 ± 124 ^h,i^	649 ± 68 ^h,i^	0.140 ± 0.005 ^g,h^	660 ± 47 ^d,e^
PSSPKC	4894 ± 87 ^h,i^	587 ± 20 ^g,h,i^	0.120 ± 0.001 ^a,b,c^	1180 ± 10 ^g,h^
PSSPXG	1258 ± 85 ^a,b,c,d,e^	171 ± 14 ^a,b,c^	0.137 ± 0.002 ^b,c,d,e^	437 ± 26 ^b,c,d^
PSPPAG	2208 ± 95 ^d,e,f,g^	251 ± 8 ^c,d,e^	0.124 ± 0.008 ^b,c,d,e^	447 ± 31 ^a,b,c,d^
PSPPCMC	2501 ± 26 ^f,g^	431 ± 6 ^d,e,f,g^	0.172 ± 0.003 ^f,g,h^	658 ± 7 ^d,e^
PSPPKC	5591 ± 108 ^i^	597 ± 14 ^g,h,i^	0.110 ± 0.010 ^a,b^	1875 ± 19 ^i,j^
PSPPXG	2410 ± 115 ^e,f,g^	397 ± 18 ^d,e,f^	0.165 ± 0.002 ^d,e,f,g^	628 ± 21 ^c,d,e^
PSWPAG	527 ± 11 ^b^	93 ± 5 ^a^	0.176 ± 0.006 ^a,b^	189 ± 7 ^a,b^
PSWPCMC	1751 ± 31 ^c,d,e,f,g^	299 ± 11 ^c,d,e^	0.171 ± 0.013 ^a,b,c,d^	380 ± 17 ^a,b,c,d^
PSWPKC	4446 ± 67 ^h,i^	572 ± 58 ^f,g,h,i^	0.128 ± 0.002 ^f,g^	1269 ± 48 ^f,g^
PSWPXG	1258 ± 42 ^b,c,d,e^	171 ± 8 ^a,b,c^	0.136 ± 0.014 ^a,b,c^	303 ± 42 ^a,b,c^
PS15	134 ± 23 ^a^	52 ± 8 ^a^	0.390 ± 0.020 ^i^	279 ± 15 ^a,b,c^
PS20	2569 ± 133 ^f,g^	254 ± 17 ^c,d,e^	0.099 ± 0.010 ^a^	2279 ± 69 ^j^

Results are displayed as mean values ± SD in triplicate. Different lowercase letters indicate significant differences among the samples concerning each starch type at *p* < 0.05.

**Table 2:** Steady shear power-law parameters for all the food ink formulations.

Samples	k (Pa*s^n^)	n (Dimensionless)	R^2^
CSFPAG	243 ± 9 ^b,c,d^	0.22 ± 0.05 ^a,b,c,d^	1.00
CSFPCMC	321 ± 7 ^c,d,e^	0.17 ± 0.03 ^a,b,c^	0.98
CSFPKC	1309 ± 18 ^j^	0.13 ± 0.02 ^a^	0.97
CSFPXG	156 ± 5 ^a,b,c^	0.21 ± 0.04 ^a,b,c,d^	0.97
CSRPAG	63 ± 4 ^a,b^	0.29 ± 0.09 ^c,d,e^	0.98
CSRPCMC	115 ± 6 ^a,b^	0.32 ± 0.05 ^e^	0.98
CSRPKC	513 ± 11 ^f,g,h^	0.31 ± 0.05 ^d,e^	0.98
CSRPXG	57 ± 2 ^a^	0.26 ± 0.01 ^b,c,d,e^	1.00
CSSPAG	541 ± 15 ^g,h,i^	0.2 ± 0.08 ^a,b,c,d,e^	0.98
CSSPCMC	648 ± 13 ^h,i^	0.26 ± 0.01 ^b,c,d,e^	0.99
CSSPKC	1242 ± 26 ^j^	0.21 ± 0.06 ^a,b,c^	0.98
CSSPXG	219 ± 7 ^a,b,c,d^	0.25 ± 0.01 ^a,b,c,d,e^	0.97
CSPPAG	167 ± 9 ^a,b,c^	0.25 ± 0.04 ^b,c,d,e^	0.99
CSPPCMC	126 ± 11 ^a,b^	0.32 ± 0.03 ^d,e^	0.99
CSPPKC	653 ± 15 ^g,h,i^	0.35 ± 0.03 ^e^	0.99
CSPPXG	237 ± 14 ^a,b,c,d^	0.29 ± 0.04 ^c,d,e^	0.96
CSWPAG	482 ± 13 ^e,f,g^	0.19 ± 0.08 ^b,c,d,e^	0.96
CSWPCMC	320 ± 12 ^c,d,e^	0.26 ± 0.06 ^b,c,d,e^	0.97
CSWPKC	737 ± 23 ^i^	0.25 ± 0.02 ^b,c,d,e^	0.99
CSWPXG	203 ± 11 ^a,b,c^	0.23 ± 0.08 ^b,c,d,e^	0.98
CS15	350 ± 2 ^c,d,e^	0.39 ± 0.01 ^f^	0.98
CS20	577 ± 7 ^g,h,i^	0.32 ± 0.02 ^d,e^	0.98
PSFPAG	243 ± 15 ^a,b,c,d^	0.22 ± 0.05 ^a,b,c^	0.99
PSFPCMC	650 ± 20 ^j^	0.14 ± 0.05 ^a^	0.96
PSFPKC	967 ± 25 ^k^	0.23 ± 0.02 ^a,b,c^	0.99
PSFPXG	159 ± 5 ^a^	0.17 ± 0.01 ^a^	0.98
PSRPAG	196 ± 12 ^a,b,c^	0.18 ± 0.04 ^a,b^	0.99
PSRPCMC	364 ± 13 ^d,e,f^	0.19 ± 0.02 ^a,b^	0.99
PSRPKC	485 ± 19 ^g,h,i^	0.21 ± 0.05 ^a,b,c^	0.98
PSRPXG	151 ± 6 ^a^	0.25 ± 0.06 ^a,b,c^	0.99
PSSPAG	519 ± 14 ^h,i^	0.19 ± 0.01 ^a,b^	0.99
PSSPCMC	673 ± 15 ^j^	0.25 ± 0.03 ^a,b,c^	0.99
PSSPKC	912 ± 25 ^k^	0.21 ± 0.08 ^a,b,c^	0.97
PSSPXG	180 ± 11 ^a,b^	0.20 ± 0.05 ^a,b,c^	0.97
PSPPAG	375 ± 3 ^e,f,g^	0.21 ± 0.03 ^a,b,c^	0.99
PSPPCMC	457 ± 18 ^f,g,h,i^	0.23 ± 0.03 ^a,b,c^	0.98
PSPPKC	650 ± 25 ^j^	0.18 ± 0.01 ^a,b^	0.97
PSPPXG	171 ± 5 ^a,b^	0.22 ± 0.02 ^a,b,c^	0.99
PSWPAG	443 ± 18 ^f,g,h^	0.36 ± 0.09 ^e^	1.00
PSWPCMC	289 ± 9 ^b,c,d,e^	0.24 ± 0.07 ^a,b,c^	1.00
PSWPKC	555 ± 24 ^h,i,j^	0.28 ± 0.01 ^b,c,d^	0.99
PSWPXG	319 ± 12 ^c,d,e^	0.23 ± 0.10 ^a,b,c^	0.99
PS15	142 ± 8 ^a^	0.17 ± 0.01 ^a,b^	0.97
PS20	389 ± 11 ^e,f,g^	0.16 ± 0.03 ^a^	0.99

Results are displayed as mean values ± SD in triplicate. Different lowercase letters indicate significant differences among samples concerning each starch type at *p* < 0.05. “k” and “n” are constant values and R2 is the determination coefficient.

**Table 3:** Thermal properties for the corn-starch-based ink formulations.

Samples	T_o_ (°C)	T_end_ (°C)	T_p_ (°C)	ΔH (J/g)
CSFPXG	71.1 ± 0.5 ^c,d,e^	81.7 ± 0.1 ^b,c,d^	75.7 ± 0.4	1.8 ± 0.3 ^a^
CSFPCMC	71.1 ± 0.2 ^d,e^	81.8 ± 0.1 ^b,c,d^	76.0 ± 0.1	1.8 ± 0.1 ^a^
CSFPAG	72.0 ± 1.7 ^e^	79.7 ± 1.6 ^a,b^	75.3 ± 0.7	1.4 ± 0.1 ^a^
CSFPKC	70.9 ± 0.3 ^b,c,d,e^	82.1 ± 0.7 ^c,d^	75.9 ± 0.1	2.1 ± 0.5 ^a^
CSWPXG	70.3 ± 0.1 ^a,b,c,d^	82.3 ± 1.4 ^c,d^	74.9 ± 0.1	1.9 ± 0.6 ^a^
CSWPCMC	70.5 ± 0.2 ^a,b,c,d^	82.6 ± 0.6 ^c,d^	75.8 ± 0.4	2.2 ± 0.4 ^a^
CSWPAG	69.6 ± 0.2 ^a,b^	81.0 ± 0.4 ^a,b,c^	74.8 ± 0.3	2.2 ± 0.3 ^a^
CSWPKC	70.5 ± 0.1 ^a,b,c,d^	82.1 ± 0.1 ^b,c,d^	75.6 ± 0.2	2.3 ± 0.2 ^a^
CSRPXG	69.4 ± 0.1 ^a^	79.3 ± 0.3 ^a^	73.8 ± 0.1	1.6 ± 0.4 ^a^
CSRPCMC	70.2 ± 0.1 ^a,b,c,d^	81.0 ± 0.3 ^a,b,c^	75.0 ± 0.2	1.8 ± 0.1 ^a^
CSRPAG	69.2 ± 1.1 ^a,b,c^	79.6 ± 1.3 ^a,b,c^	73.8 ± 1.3	1.5 ± 0.4 ^a^
CSRPKC	70.5 ± 0.1 ^a,b,c,d^	80.7 ± 0.3 ^a,b,c^	75.0 ± 0.2	1.9 ± 0.3 ^a^
CSPPXG	71.1 ± 0.3 ^b,c,d,e^	82.2 ± 0.4 ^c,d^	75.9 ± 0.4	1.8 ± 0.3 ^a^
CSPPCMC	70.8 ± 0.2 ^a,b,c,d,e^	82.1 ± 0.4 ^c,d^	75.8 ± 0.2	2.0 ± 0.3 ^a^
CSPPAG	69.7 ± 0.3 ^a,b,c^	80.8 ± 0.2 ^a,b,c^	74.1 ± 0.3	1.0 ± 0.3 ^a^
CSPPKC	70.7 ± 0.1 ^a,b,c,d,e^	81.9 ± 0.2 ^b,c,d^	75.5 ± 0.2	2.0 ± 0.2 ^a^
CSSPXG	70.9 ± 0.1 ^b,c,d,e^	81.6 ± 0.1 ^b,c,d^	75.4 ± 0.2	1.6 ± 0.6 ^a^
CSSPCMC	70.6 ± 0.2 ^a,b,c,d,e^	81.6 ± 0.1 ^a,b,c,d^	75.6 ± 0.1	1.6 ± 0.1 ^a^
CSSPAG	70.0 ± 0.6 ^a,b,c,d^	80.9 ± 0.3 ^a,b,c^	74.6 ± 0.5	1.7 ± 0.1 ^a^
CSSPKC	70.7 ± 0.2 ^a,b,c,d,e^	82.5 ± 0.1 ^c,d^	75.8 ± 0.1	2.3 ± 0.2 ^a^
Csnative	71.2 ± 0.2^d,e^	83.5 ± 0.1 ^d^	75.5 ± 0.4	12.6 ± 1.1 ^b^

Results are displayed as mean values ± SD in triplicate. Different lowercase letters indicate significant differences among samples at *p* < 0.05.

**Table 4:** Thermal properties for the potato-starch-based ink formulations.

Samples	T_o_ (°C)	T_end_ (°C)	T_p_ (°C)	ΔH (J/g)
PSFPXG	62.6 ± 0.1 ^d,e^	73.2 ± 0.3 ^b^	67.0 ± 0.1 ^e,f^	2.5 ± 0.1 ^a,b^
PSFPCMC	62.8 ± 0.1 ^e^	73.5 ± 0.2 ^b^	67.4 ± 0.1 ^f^	2.5 ± 0.2 ^a,b^
PSFPAG	61.7 ± 0.7 ^a,b,c,d,e^	71.1 ± 1.7 ^a,b^	65.5 ± 1.2 ^a,b,c,d^	1.9 ± 1.2 ^a,b^
PSFPKC	62.5 ± 0.1 ^d,e^	72.4 ± 0.2 ^a,b^	66.8 ± 0.1 ^c,d,e,f^	2.7 ± 0.1 ^a,b^
PSWPXG	62.4 ± 0.2 ^c,d,e^	72.4 ± 0.3 ^a,b^	66.8 ± 0.2 ^d,e,f^	2.1 ± 0.1 ^a,b^
PSWPCMC	61.7 ± 0.2 ^a,b,c,d,e^	73.9 ± 1.8 ^b^	66.2 ± 0.1 ^a,b,c,d,e,f^	2.4 ± 0.3 ^a,b^
PSWPAG	61.6 ± 0.2 ^a,b,c,d^	72.0 ± 0.9 ^a,b^	66.1 ± 0.4 ^a,b,c,d,e,f^	2.7 ± 0.3 ^a,b^
PSWPKC	61.8 ± 0.2 ^a,b,c,d,e^	73.2 ± 1.1 ^b^	66.3 ± 0.2 ^b,c,d,e,f^	3.2 ± 0.5 ^b^
PSRPXG	61.8 ± 0.1 ^a,b,c,d,e^	71.3 ± 0.1 ^a,b^	66.1 ± 0.1 ^a,b,c,d,e^	2.3 ± 0.2 ^a,b^
PSRPCMC	62.2 ± 0.2 ^c,d,e^	72.2 ± 0.4 ^a,b^	66.6 ± 0.2 ^b,c,d,e,f^	2.3 ± 0.2 ^a,b^
PSRPAG	60.8 ± 0.9 ^a^	73.4 ± 1.0 ^b^	64.9 ± 1.1 ^a^	1.7 ± 0.9 ^a^
PSRPKC	62.2 ± 0.2 ^c,d,e^	71.6 ± 0.2 ^a,b^	66.3 ± 0.2 ^b,c,d,e,f^	2.2 ± 0.1 ^a,b^
PSPPXG	62.1 ± 0.2 ^c,d,e^	72.7 ± 0.6 ^a,b^	66.7 ± 0.3 ^b,c,d,f^	2.4 ± 0.1 ^a,b^
PSPPCMC	61.9 ± 0.1 ^a,b,c,d,e^	72.0 ± 0.4 ^a,b^	66.5 ± 0.2 ^b,c,d,f^	2.3 ± 0.4 ^a,b^
PSPPAG	61.3 ± 0.6 ^a,b,c^	71.0 ± 1.1 ^a,b^	65.3 ± 0.4 ^a,b^	2.1 ± 1.1 ^a,b^
PSPPKC	61.7 ± 0.2 ^a,b,c,d^	72.3 ± 0.9 ^a,b^	66.4 ± 0.2 ^b,c,d.e,f^	2.6 ± 0.1 ^a,b^
PSSPXG	61.8 ± 0.1 ^a,b,c,d,e^	72.1 ± 0.2 ^a,b^	66.5 ± 0.1 ^b,c,d,e,f^	2.5 ± 0.4 ^a,b^
PSSPCMC	62.0 ± 0.2 ^c,d,e^	72.3 ± 0.2 ^a,b^	66.7 ± 0.2 ^b,c,d,e,f^	2.4 ± 0.1 ^a,b^
PSSPAG	60.8 ± 0.9 ^a^	72.5 ± 1.1 ^a,b^	65.4 ± 0.8 ^a,b^	1.9 ± 0.4 ^a^
PSSPKC	62.0 ± 0.2 ^b,c,d,e^	72.1 ± 0.5 ^a,b^	66.4 ± 0.3 ^b,c,d,e,f^	2.2 ± 0.2 ^a,b^
PSnative	64.8 ± 0.1 ^f^	77.7 ± 0.1 ^a^	69.4 ± 0.2 ^g^	15.9 ± 0.6 ^c^

Results are displayed as mean values ± SD in triplicate. Different lowercase letters in the same column show a significant difference among samples at *p* < 0.05.

## Data Availability

The original contributions presented in this study are included in the article/Supplementary Materials. Further inquiries can be directed to the corresponding author.

## Supplementary Materials

The following supporting information can be downloaded at: https://www.mdpi.com/article/10.3390/gels11080574/s1, Table S1. Customized 3D printer system set-up; Table S2. Composition of food inks with corn starch substrate and 3D printing suitability; Table S3. Composition of food inks with potato starch substrate and 3D printing suitability; Figure S1: Flow curves of for ink formulations with best printability features; Figure S2: Flow curves of for ink formulations with best printability features; Figure S3: Shear stress distribution in the syringe wall of best printing ink formulations; Figure S4: FTIR spectra corn starch (CS), k-carrageenan (KC), potato starch (PS); Figure S5: FTIR spectra protein (PP), fava protein (FP), rice protein (RP), soya protein (SP) and whey protein (WP).

## Abbreviations

The following abbreviations are used in this manuscript:

CS	corn starch
PS	potato starch
AG	arabic gum
XG	xanthan gum
KC	k-carrageenan
CMC	carboxy methyl cellulose
FP	fava bean protein
WP	whey protein
SP	soya protein
PP	pea protein
RP	rice protein
η	viscosity
G′	storage modulus
G″	loss modulus
tan δ	phase angle δ
K	consistency index
n	flow index
τy	yield stress
T_on_	gelatinization onset temperature
T_p_	gelatinization peak temperature
T_end_	gelatinization endset temperature
ΔH	enthalpy of gelatinization
CSFPAG	corn starch, fava bean protein, arabic gum
CSFPCMC	corn starch, fava bean protein, carboxy methyl cellulose
CSFPKC	corn starch, fava bean protein, k-carrageenan
CSFPXG	corn starch, fava bean protein, xanthan gum
CSRPAG	corn starch, rice protein, arabic gum
CSRPCMC	corn starch, rice protein, carboxy methyl cellulose
CSRPKC	corn starch, rice protein, k-carrageenan
CSRPXG	corn starch, rice protein, xanthan gum
CSSPAG	corn starch, soya protein, arabic gum
CSSPCMC	corn starch, soya protein, carboxy methyl cellulose
CSSPKC	corn starch, soya protein, k-carrageenan
CSSPXG	corn starch, soya protein, xanthan gum
CSPPAG	corn starch, pea protein, arabic gum
CSPPCMC	corn starch, pea protein, carboxy methyl cellulose
CSPPKC	corn starch, pea protein, k-carrageenan
CSPPXG	corn starch, pea protein, xanthan gum
CSWPAG	corn starch, whey protein, arabic gum
CSWPCMC	corn starch, whey protein, carboxy methyl cellulose
CSWPKC	corn starch, whey protein, k-carrageenan
CSWPXG	corn starch, whey protein, xanthan gum
CS15	corn starch 15% *w*/*v*
CS20	corn starch 20% *w*/*v*
PSFPAG	potato starch, fava bean protein, arabic gum
PSFPCMC	potato starch, fava bean protein, carboxy methyl cellulose
PSFPKC	potato starch, fava bean protein, k-carrageenan
PSFPXG	potato starch, fava bean protein, xanthan gum
PSRPAG	potato starch, rice protein, arabic gum
PSRPCMC	potato starch, rice protein, carboxy methyl cellulose
PSRPKC	potato starch, rice protein, k-carrageenan
PSRPXG	potato starch, rice protein, xanthan gum
PSSPAG	potato starch, soya protein, arabic gum
PSSPCMC	potato starch, soya protein, carboxy methyl cellulose
PSSPKC	potato starch, soya protein, k-carrageenan
PSSPXG	potato starch, soya protein, xanthan gum
PSPPAG	potato starch, pea protein, arabic gum
PSPPCMC	potato starch, pea protein, carboxy methyl cellulose
PSPPKC	potato starch, pea protein, k-carrageenan
PSPPXG	potato starch, pea protein, xanthan gum
PSWPAG	potato starch, whey protein, arabic gum
PSWPCMC	potato starch, whey protein, carboxy methyl cellulose
PSWPKC	potato starch, whey protein, k-carrageenan
PSWPXG	potato starch, whey protein, xanthan gum
PS15	potato starch 15% *w*/*v*
PS20	potato starch 20% *w*/*v*
