# Supplementary material for: The Development and Optimization of Extrusion-Based 3D Food Printing Inks Using Composite Starch Gels Enriched with Various Proteins and Hydrocolloids
Source: Gels. 2025 Jul 23;11(8):574. doi: 10.3390/gels11080574 (PMC12385182; doi:10.3390/gels11080574)
Supplement: Supplementary file 1 [file gels-11-00574-s001.zip › gels-3729562-supplementary.pdf]

The development and optimization of extrusion-based 3D food printing inks using composite starch gels enriched with various proteins and hydrocolloids.

Evgenia N. Nikolaou [1*], Eftychios Apostolidis [1], Irene K. Nikolidaki [1], Evangelia D. Karvela [1], Athena Stergiou [1], Thomas Kourtis [1], and Vaios T. Karathanos [1]

[1] Harokopion University, Department of Science of Dietetics-Nutrition, 70, El. Venizelou, 17671, Kallithea, Athens, Greece
* Correspondence: evgenia@hua.gr; Tel.: (optional; include country code; if there are multiple corresponding authors, add author initials)

Supplementary

Table S1. Customized 3D printer system set-up

3D Printing Technology	Title 2
3D Printing Technology	Material Extrusion
Max Nozzle Temperature	180 °C (max 250 °C)
Max Printing Platform Temperature	110 °C
Printing Speed	100 mm/s (max. material-dependent)
Working Capacity	170 x 170 x 170 mm
Platform Material	Silicon (food interface approved) removable
Syringe Volume	60 cc (max)
Nozzle Diameter	0.5 – 0.8 – 1.2 – 2.0 mm
Air Pressure	10 bars (max)
Power Consumption (Max)	350 W (air compressor not included)
Operating Voltage	24V. 15A

Table S2. Composition of food inks with corn starch substrate and 3D printing suitability

Abbreviations	Corn Starch (CS) %(w/v)	Protein Concentrate					Hydrocolloid				Print Score/ Observations
		Fava (FP) %(w/v)	Rice (RP) %(w/v)	Soya (SP) %(w/v)	Pea (PP) %(w/v)	Whey (WP) %(w/v)	k-Carrageenan (KC) %(w/v)	Arabic Gum (AG) %(w/v)	Xanthan Gum (XG) %(w/v)	CMC %(w/v)	
**CS15**	**15**										**3-Indistinct layer, extrusion difficulty** 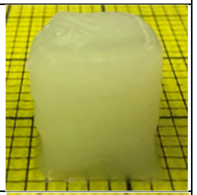
**CS20**	**20**										**2- Indistinct layer, high extrusion difficulty, intermittent printing** 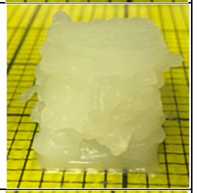
**CSFPKC**	**15**	**5**					**5**				**5-Distinct layer, self-support** 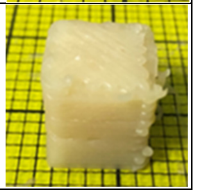
**CSFPAG**	**15**	**5**						**5**			**0-Unprintable**
**CSFPXG**	**15**	**5**							**5**		**2-Merged layering** 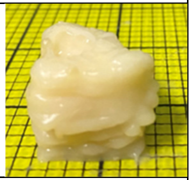
**CSFPCMC**	**15**	**5**								**5**	**2- Merged layering** 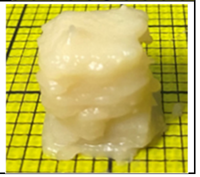

**CSRPKC**	**15**		**5**				**5**				**5-Distinct layer, self-support** 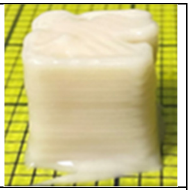
**CSRPAG**	**15**		**5**					**5**			**2-Merged layers, low viscosity, Spread** 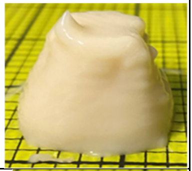
**CSRPXG**	**15**		**5**						**5**		**1-low viscosity, spread** 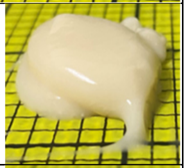
**CSRPCMC**	**15**		**5**							**5**	**2-Merged Layering** 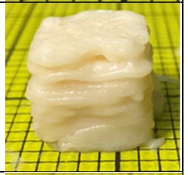
**CSSPKC**	**15**			**5**			**5**				**5-Distinct layer, self-support** 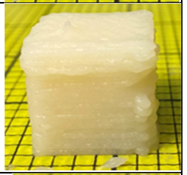
**CSSPAG**	**15**			**5**				**5**			**4- Distinct layer, flow discontinuity** 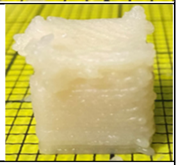
**CSSPXG**	**15**			**5**					**5**		**0-Not printable**
**CSSPCMC**	**15**			**5**						**5**	**3- Distinct layer, top-layer flow discontinuities, rough edges** 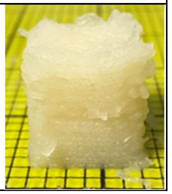

**CSPPKC**	**15**				**5**		**5**				**4-Distinct layer, medium to top layer flow discontinuity** 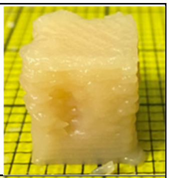
**CSPPAG**	**15**				**5**			**5**			**3-Not distinct layering, smooth extrusion** 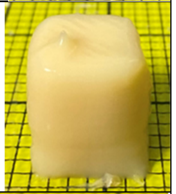
**CSPPXG**	**15**				**5**				**5**		**4-Not distinct layer** 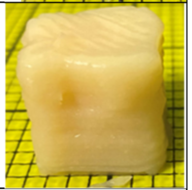
**CSPPCMC**	**15**				**5**					**5**	**2-Merged layer** 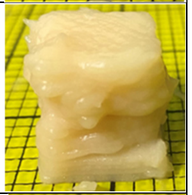
**CSWPKC**	**15**					**5**	**5**				**5-Distinct layer, self-support** 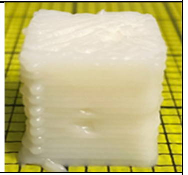
**CSWPAG**	**15**					**5**		**5**			**2-Clogging, intermittent extrusion** 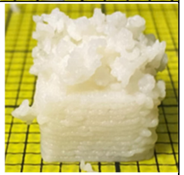
**CSWPXG**	**15**					**5**			**5**		**0-Unprintable**
**CSWPCMC**	**15**					**5**				**5**	**0-Unprintable**

Table S3. Composition of food inks with potato starch substrate and 3D printing suitability

Abbreviations	Potato Starch (%W/V)	Protein Concentrate: Fava (FP) (%w/v)	Protein Concentrate: Rice (RP) (%w/v)	Protein Concentrate: Soya (SP) (%w/v)	Protein Concentrate: Pea (PP) (%w/v)	Protein Concentrate: Whey (WP) (%w/v)	Hydrocolloid: k-Carrageenan (KC) (%w/v)	Hydrocolloid: Arabic Gum (AG) (%w/v)	Hydrocolloid: Xanthan Gum (XG) (%w/v)	Hydrocolloid: CMC (%w/v)	Print score/ Observations
**PS15**	**15**										**2-Indistinct layer, extrusion difficulty** 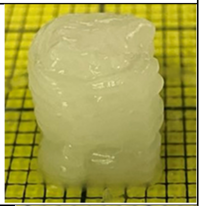
**PS20**	**20**										**1-Extrusion difficulty** 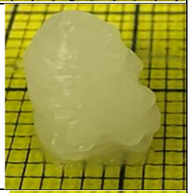
**PSFPKC**	**15**	**5**					**5**				**1- Clogging, intermittent extrusion** 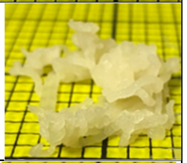
**PSFPAG**	**15**	**5**						**5**			**1- low viscosity, spread** 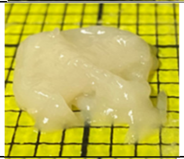
**PSFPXG**	**15**	**5**							**5**		**1- low viscosity, spread** 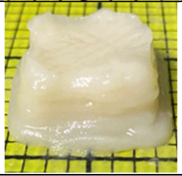
**PSFPCMC**	**15**	**5**								**5**	**0-Unprintable**

**PSRPKC**	**15**		**5**				**5**				**5- Distinct layer, self-support**	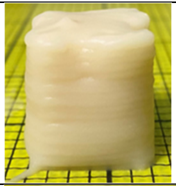
**PSRPAG**	**15**		**5**					**5**			**4-smooth extrusion, structure collapse**	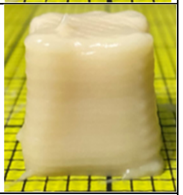
**PSRPXG**	**15**		**5**						**5**		**0-Unprintable**	
**PSRPCMC**	**15**		**5**							**5**	**0-Unprintable**	
**PSSPKC**	**15**			**5**			**5**				**4-Distinct layer, self support, high viscosity, nozzle clogging**	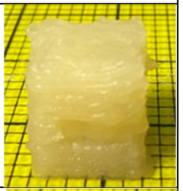
**PSSPAG**	**15**			**5**				**5**			**2-low viscosity, spreading**	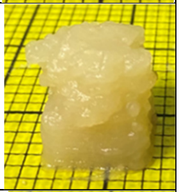
**PSSPXG**	**15**			**5**					**5**		**1- low viscosity, spreading**	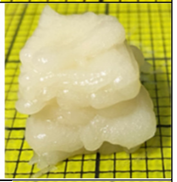
**PSSPCMC**	**15**			**5**						**5**	**3-Indistinct layer, intermittent extrusion**	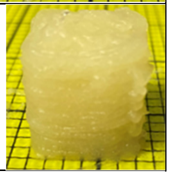

**PSPPKC**	**15**				**5**		**5**				**4-Distinct layer, intermittent extrusion**	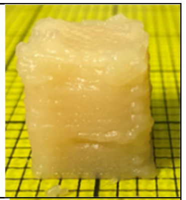
**PSPPAG**	**15**				**5**			**5**			**3-Distinct layer, nozzle clogging at top layers**	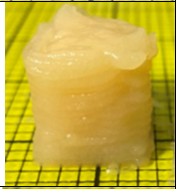
**PSPPXG**	**15**				**5**				**5**		**3-smooth extrusion, indistinct layer, structure collapse**	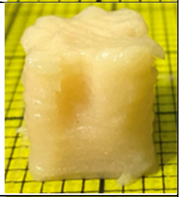
**PSPPCMC**	**15**				**5**					**5**	**4-Distinct layer, self support, intermittent extrusion**	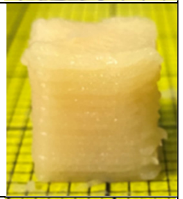
**PSWPKC**	**15**					**5**	**5**				**5-Distinct layer, self support**	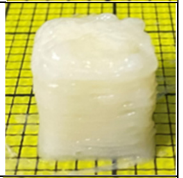
**PSWPAG**	**15**					**5**		**5**			**3-Indistinct layer, intermittent extrusion, nozzle clogging at top layers**	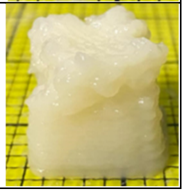

**PSWPXG**	**15**					**5**			**5**		**3-Distinct layer, nozzle clogging at top layers** 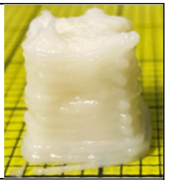
**PSWPCMC**	**15**					**5**				**5**	**0-Unprintable**

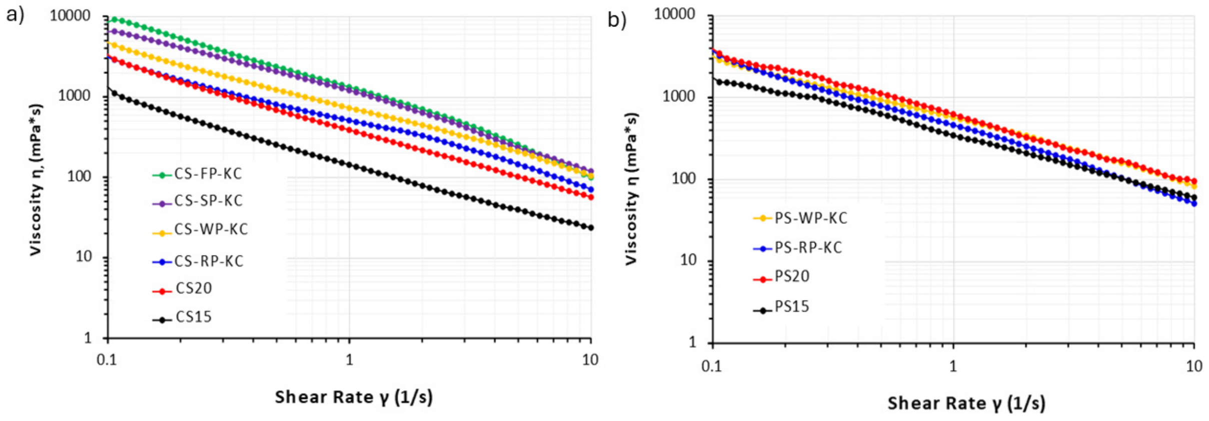

Figure S1: Flow curves of for ink formulations with best printability features

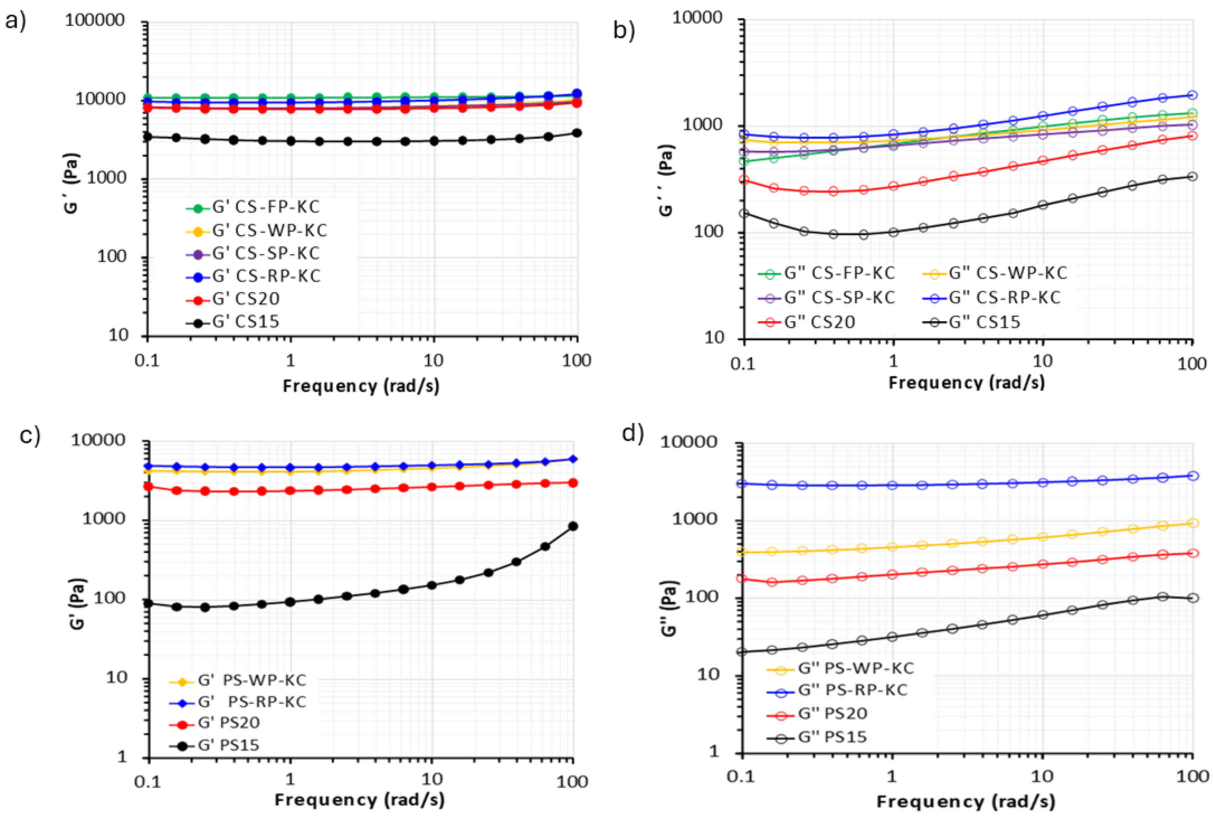

Figure S2: Flow curves of for ink formulations with best printability features

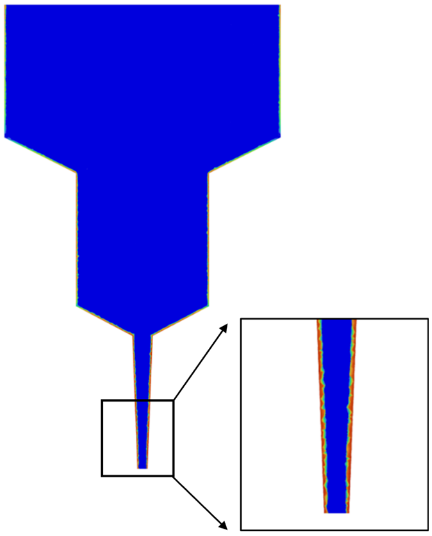

Figure S3: Shear stress distribution in the syringe wall of best printing ink formulations

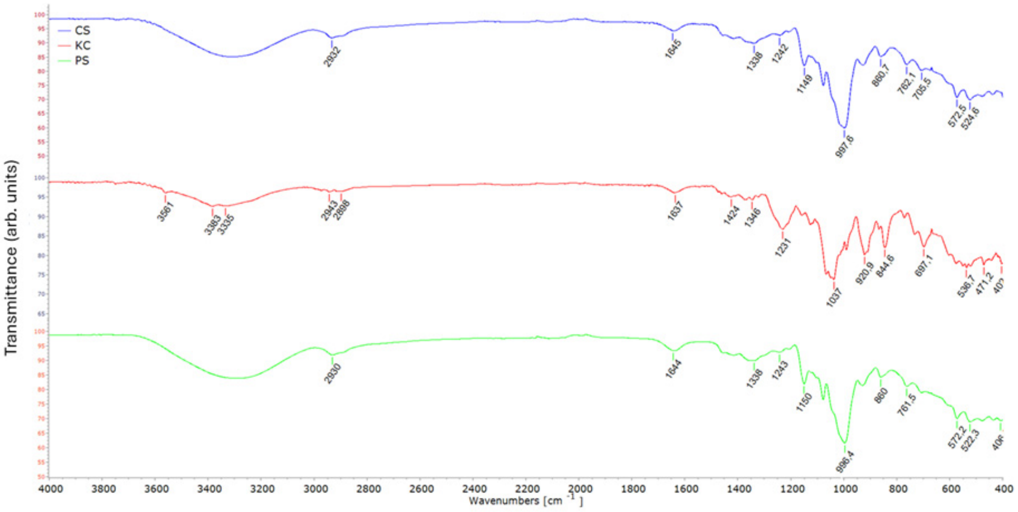

Figure S4: FTIR spectra corn starch (CS), k-carrageenan (KC), potato starch (PS)

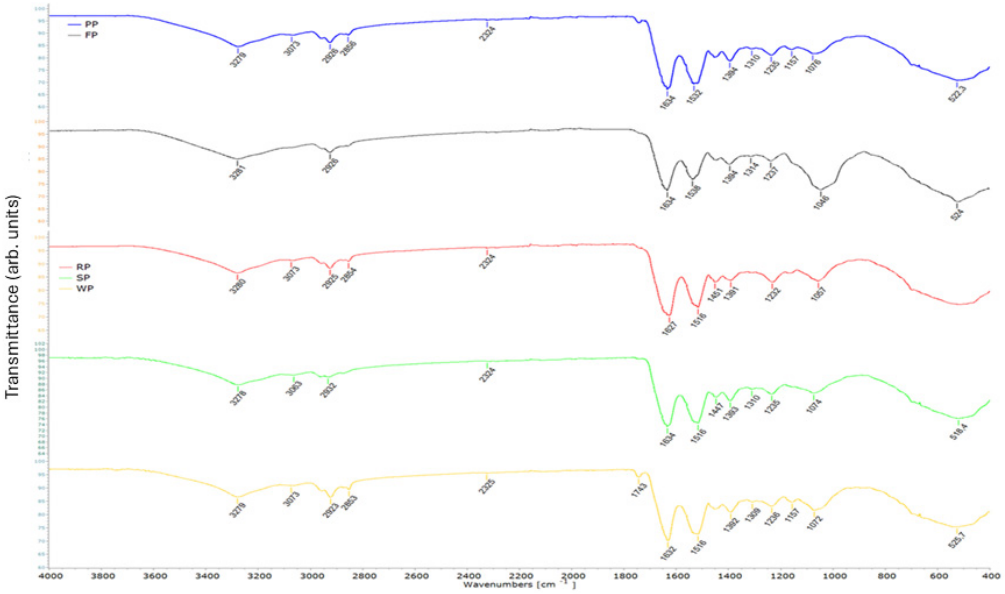

Figure S5: FTIR spectra protein (PP), fava protein (FP), rice protein (RP), soya protein (SP) and whey protein (WP
